# Enrichment of syngas-converting mixed microbial consortia for ethanol production and thermodynamics-based design of enrichment strategies

**DOI:** 10.1186/s13068-018-1189-6

**Published:** 2018-07-19

**Authors:** Antonio Grimalt-Alemany, Mateusz Łężyk, Lene Lange, Ioannis V. Skiadas, Hariklia N. Gavala

**Affiliations:** Department of Chemical and Biochemical Engineering, Technical University of Denmark, Søltofts Plads 229, 2800 Kgs. Lyngby, Denmark

**Keywords:** Enrichment, Syngas, Carbon monoxide, Mixed culture, Microbial consortia, Ethanol, Thermodynamics

## Abstract

**Background:**

The production of ethanol through the biochemical conversion of syngas, a mixture of H_2_, CO and CO_2_, has been typically studied using pure cultures. However, mixed microbial consortia may offer a series of benefits such as higher resilience and adaptive capacity, and non-sterile operation, all of which contribute to reducing the utility consumption when compared to pure culture-based processes. This work focuses on the study of strategies for the enrichment of mixed microbial consortia with high ethanologenic potential, investigating the effect of the operational conditions (pH and yeast extract addition) on both the ethanol yield and evolution of the microbial community along the enrichment process. The pH was selected as the main driver of the enrichment as it was expected to be a crucial parameter for the selection of carboxydotrophic bacteria with high ethanologenic potential. Additionally, a thermodynamic analysis of the network of biochemical reactions carried out by syngas-converting microbial consortia was performed and the potential of using thermodynamics as a basis for the selection of operational parameters favoring a specific microbial activity was evaluated.

**Results:**

All enriched consortia were dominated by the genus *Clostridium* with variable microbial diversity and species composition as a function of the enrichment conditions. The ethanologenic potential of the enriched consortia was observed to increase as the initial pH was lowered, achieving an ethanol yield of 59.2 ± 0.2% of the theoretical maximum in the enrichment at pH 5. On the other hand, yeast extract addition did not affect the ethanol yield, but triggered the production of medium-chain fatty acids and alcohols. The thermodynamic analysis of the occurring biochemical reactions allowed a qualitative prediction of the activity of microbial consortia, thus enabling a more rational design of the enrichment strategies targeting specific activities. Using this approach, an improvement of 22.5% over the maximum ethanol yield previously obtained was achieved, reaching an ethanol yield of 72.4 ± 2.1% of the theoretical maximum by increasing the initial acetate concentration in the fermentation broth.

**Conclusions:**

This study demonstrated high product selectivity towards ethanol using mixed microbial consortia. The thermodynamic analysis carried out proved to be a valuable tool for interpreting the metabolic network of microbial consortia-driven processes and designing microbial-enrichment strategies targeting specific biotransformations.

**Electronic supplementary material:**

The online version of this article (10.1186/s13068-018-1189-6) contains supplementary material, which is available to authorized users.

## Background

Over the past decades, the rising concerns about climate change and the depletion of fossil fuels, along with the ever increasing demand of transportation fuels, have led to the global implementation of policies fostering biofuel production. As a result of these policies, the biofuel market underwent a rapid expansion rising from less than 20 billion liters/year in 2001 to over 100 billion liters/year in 2011 [1]. However, the rapid growth of this market, strictly based on first generation biofuels, brought along a series of environmental and socioeconomic impacts derived from the competition with food crops such as land use change, rising food and feed prices, and poor greenhouse gas emission savings [2, 3]. Developing second-generation biofuel technologies is thus considered an important step forward as these are based on the use of non-food biomasses and waste streams as feedstock, and are expected to overcome the limitations of first-generation biofuels in terms of environmental impacts and range of exploitable feedstocks.

Among the different approaches within second-generation biofuel technologies, syngas fermentation is one of the most promising as it combines the benefits of both thermochemical and biochemical biomass conversion processes. Typically, this process comprises thermochemical conversion of the biomass through gasification into synthesis gas, a mixture of mainly H_2_, CO_2_ and CO, followed by its biological conversion into a variety of chemicals and fuels [4–7]. The fermentation of syngas is carried out by anaerobic acetogenic bacteria, which are able to use both CO and H_2_/CO_2_ as the sole carbon and energy source through the Wood–Ljungdahl pathway. So far, mostly pure cultures have been employed in syngas fermentation processes, with the most common wild-type strains being *C. autoethanogenum* [8], *C. ljungdahlii* [9], *C. ragsdalei* [10] and *C. carboxidivorans* [11]. However, several studies have reported that microbial growth on CO and H_2_/CO_2_ can be significantly inhibited by the impurities of syngas [12, 13], which may result in decreased productivity of syngas fermentation systems and/or higher raw syngas clean-up requirements. Another limitation is the fact that sterile operation is necessary to avoid a possible microbial contamination of the monoculture, which increases the energy input requirements.

A possible alternative to overcome these limitations is to use open-mixed microbial consortia. Developing microbial consortia-driven processes may allow reducing the cleaning requirements of raw syngas as they present a higher resilience and adaptive capacity due to their microbial diversity [14]. Additionally, sterilization is not necessary when using microbial consortia [15], which contributes to reducing the utility consumption. Nonetheless, their higher complexity often entails an inadequate understanding of the microbial interactions within the consortium, resulting in limited process control. Low product selectivity is another challenge generally encountered in microbial consortia-driven processes, ultimately lowering the yield of the desired product.

Although the use of open-mixed microbial consortia in syngas fermentation processes is still rather limited compared to pure cultures, the potential of microbial consortia for producing H_2_ [16], CH_4_ [17], carboxylic acids [18–20] and higher alcohols [21] has been demonstrated in a number of studies. The production of solvents through the reduction of carboxylic acids using either syngas or H_2_ as electron donor has also been studied [22, 23]. However, scientific literature on the production of ethanol by mixed microbial consortia using syngas as the sole carbon source is scarce, with only two studies showing a significant ethanologenic potential. Singla et al. [24] were the first demonstrating a high ethanol production using an enriched mixed culture from the culture collection of TERI University. In their study, the operating conditions were optimized for maximizing the ethanol yield in batch experiments, obtaining a maximum concentration of 1.54 and 0.9 g/l of ethanol and acetic acid, respectively. In turn, Ganigué et al. [21] studied the enrichment of a pre-acclimatized open-mixed microbial consortium using 2-bromoethanesulfonate for inhibiting methanogenic activity, where significant amounts of ethanol, butanol and hexanol were produced through syngas fermentation and chain elongation. Nevertheless, a dedicated study on enrichment of mixed microbial consortia aiming at enhancing the product selectivity towards ethanol has not been conducted yet.

This work focuses on the study of selective enrichment strategies for developing microbial consortia with high ethanologenic potential, laying special emphasis on the effect of the enrichment conditions on both the ethanol yield and selectivity, and the evolution of the microbial community along the enrichment process. Additionally, the thermodynamics of the network of biochemical reactions of microbial consortia is evaluated based on the Gibbs free energy change at different enrichment conditions with the perspective of using thermodynamics as a tool for developing selective enrichment strategies targeting specific biotransformations.

## Methods

### Inoculum source

The inoculum used was a combination of two different types of anaerobic sludge collected from the Lundtofte wastewater treatment plant (Denmark) and from a lab-scale anaerobic digester fed with manure (at Chemical and Biochemical Engineering Department, Technical University of Denmark). The inoculum was prepared by mixing the two anaerobic sludges in equal amounts (50/50 v/v) and adjusting the pH to 6 with 1 M HCl while flushing with N_2_ to ensure anaerobic conditions.

Unless otherwise stated, the starting inoculum used in the enrichments (see “Enrichment experiments and conditions”) underwent a heat-shock treatment to suppress the methanogenic activity. The heat-shock treatment was carried out by heating the mixture of anaerobic sludges up to 90 °C for 15 min while flushing with N_2_.

### Growth medium composition

A modified basal anaerobic (BA) medium was used in all experiments, which consisted of six stock solutions containing phosphate buffer, vitamins, trace elements, salts, chelating agents and reducing agents. The stock solutions had the following composition: solution A (NH_4_Cl, 100 g/l; NaCl, 10 g/l; MgCl_2_∙6H_2_O, 10 g/l; CaCl_2_∙2H_2_O, 5 g/l), vitamins solution according to Wolin et al. [25], trace metal solution (FeCl_2_∙4H_2_O, 2000 mg/l; H_3_BO_3_, 50 mg/l; ZnCl_2_, 50 mg/l; CuCl_2_, 30 mg/l; MnCl_2_∙4 H_2_O, 50 mg/l; (NH_4_)_6_Mo_7_O_24_∙4 H_2_O, 50 mg/l; AlCl_3_, 50 mg/l; CoCl_2_∙6H_2_O, 50 mg/l; NiCl_2_, 50 mg/l; Na_2_SeO_3_∙5H_2_O, 100 mg/l; Na_2_WO_4_∙2H_2_O, 60 mg/l), chelating agent solution (Nitrilotriacetic acid, 1 g/l) and reducing agent solution (Na_2_S∙9H_2_O, 25 g/l).

The medium was prepared by adding 10 ml/l of solution A, 1 ml/l of trace metal solution, 10 ml/l of vitamins solution, 10 ml/l of reducing agent solution, 20 ml/l of chelating agent solution and distilled water up to 1 l. The pH was adjusted with phosphate buffer (50 mM) using three stock solutions (K_2_HPO_4_∙3H_2_O, 200 g/l; KH_2_PO_4_, 136 g/l; H_3_PO_4_, 98 g/l). When relevant, yeast extract (YE) and sodium acetate were added to the medium with a final concentration of 0.5 g/l and 20 mM, respectively.

### Enrichment experiments and conditions

All enrichment experiments were performed in 330 ml serum flasks with an active volume of 100 ml and an inoculum size of 15% v/v (15 ml). The medium (85 ml) was added to the flasks and was flushed with H_2_ to create anaerobic conditions. After the flasks were sealed with rubber stoppers and screw plugs, the remaining gases (CO and CO_2_) were added up to a total pressure of 2 atm prior to inoculation using a precision pressure indicator (model CPH6400, WIKA, Germany). All gases used had purity above 99.9%, and were purchased from AGA (Denmark). After inoculation, the total initial pressure increased to 2.14 atm and the final gas composition of the headspace corresponded to approximately 10.1 mmol of H_2_, 4.5 mmol of CO and 5.5 mmol of CO_2_ at 25 °C. The fermentation flasks were incubated in the dark at 37 °C and 100 rpm. Control experiments were performed at the same incubation conditions with no addition of gaseous substrates, adjusting the gaseous composition of the headspace to 1.44 atm of N_2_ and 0.56 atm of CO_2_ prior to inoculation.

**Fig. 1:**
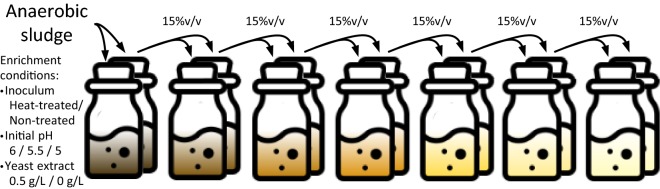
Scheme of the microbial enrichment in batch cultures

A batch-enrichment technique was used in all enrichment series, consisting in the successive transfer of a fraction of the fermented broth (used as inoculum) into a new flask with fresh medium and substrate (Fig. 1). Each enrichment series comprised a total of six transfers and seven fermentations using a 15% v/v inoculum size for each transfer. The enrichments were performed in duplicates with the best performing one being used as inoculum for the next transfer. The selection of the individual duplicate to be transferred was based on the ethanol yield, i.e., only the best ethanol-producing consortium was transferred to the next set of duplicates. The enrichment strategies were based on the pH conditions and the presence of syngas as the main drivers for selecting the most effective and efficient ethanologenic consortia. Yeast extract was also added to selected enrichment series to improve growth at low pH conditions. The enrichment conditions used, including starting inoculum, pH, substrates and nutrient supplements, are given in Table 1.

**Table 1:** Enrichment conditions based on initial pH and initial addition of acetate

Enrichment series	Inoculum treatment	Initial pH	Syngas composition (%H_2_, %CO, %CO_2_)	Co-substrate	YE (g/l)
HT6	Heat-shock	5.95 ± 0.06	50%, 22.2%, 27.8%	–	–
HT5.5	Heat-shock	5.51 ± 0.09	50%, 22.2%, 27.8%	–	–
HT5	Heat-shock	5.05 ± 0.02	50%, 22.2%, 27.8%	–	–
HT5.5YE	Heat-shock	5.43 ± 0.13	50%, 22.2%, 27.8%	–	0.5
HT5YE	Heat-shock	5.02 ± 0.10	50%, 22.2%, 27.8%	–	0.5
NT5YE	Non-treated	5.04 ± 0.13	50%, 22.2%, 27.8%	–	0.5
HT5YE-Ac	Heat-shock	4.99 ± 0.08	50%, 22.2%, 27.8%	NaCH_3_COO (20 mM)	0.5

The pH was not stable during the course of the fermentations

The enrichment series HT5YE was interrupted at transfer T4 and presented a significant loss of solventogenic activity upon resuming the enrichment. Thus, this enrichment was extended for one more transfer to confirm the recovery of the previous activity.

Samples for the determination of the metabolites concentration and yield in each transfer were taken at the beginning of the experiments and after the culture reached the stationary phase. Samples for microbial growth determination were taken from transfer T3, when the solids from the anaerobic sludge were completely diluted and did not interfere with the absorbance of the fermentation broth. As it was not possible to measure microbial growth during the first transfers of the enrichments, the distinction between exponential and stationary growth phase was based on the profile of the consumption of H_2_ and CO over time.

### DNA extraction, sequencing and microbial population analysis

For analysis of microbial composition, selected fermentation steps during enrichments were sampled at both exponential and stationary growth phase. 5 ml of culture was spun down and genomic DNA was isolated using DNeasy Blood & Tissue Kit, following manufacturer recommendations for Gram-positive bacteria (Qiagen, Copenhagen).

The DNA sample was submitted to Macrogen Inc. (Korea) for 16S rRNA amplicon library preparation and sequencing using Illumina Miseq instrument (300 bp paired-end sequencing). Amplification of V3 and V4 region of 16S rRNA gene was done with Pro341F 5′-CCTACGGGNBGCASCAG-3′ and Pro805R 5′-GACTACNVGGGTATCTAATCC-3′ [26]. Sequences containing primers were trimmed with cutadapt and all other reads filtered out [27]. Subsequently, filtering, generation of operational taxonomic units (OTUs) and mapping of reads to OTUs were performed using the UPARSE/unoise3 pipeline [28]. Taxonomy was assigned to OTUs using SINTAX and NCBI database of 18421 16S ribosomal RNA sequences from NCBI RefSeq Targeted Loci Project [29]. Subsequently, OTU table was corrected using the UNCROSS algorithm [30], normalized with respect to 16S copy number and primer mismatches with UNBIAS algorithm [31]. Each sample was normalized to the depth of the sample with least counts. OTUs with overall count less than 100 were filtered out and sample data from available replicate runs have been collapsed based on the mean count. Downstream analyses were performed with Phyloseq R [32] package and MicrobiomeAnalyst web service, available at http://www.microbiomeanalyst.ca/ [33]. Fitting of environmental variables onto ordination plots was performed with R Vegan package [34].

### Analytical methods

The gaseous composition of the headspace (H_2_, CO, CO_2_ and CH_4_) was determined using a gas chromatograph (model 8610C, SRI Instruments, USA) equipped with a thermal conductivity detector and two packed columns, a 6′ × 1/8″ Molsieve 13× column and a 6′ × 1/8″ silica gel column connected in series through a rotating valve. The column temperature was maintained at 65 °C for 3 min, followed by a temperature ramp of 10 °C/min to 95 °C and 24 °C/min from 95 to 140 °C. Gaseous samples of 50 µl were collected with a gas-tight syringe (model 1750SL, Hamilton). Volatile fatty acids (VFA) (acetate, propionate, iso-butyrate, butyrate and caproate) and alcohols (ethanol and 1-butanol) were determined using a high performance liquid chromatograph (Shimadzu, USA) equipped with a refractive index detector and an Aminex HPX-87H column (Bio-Rad, USA) at 63 °C. A solution of 12 mM H_2_SO_4_ was used as eluent at a flow rate of 0.6 ml/min. Volatile Suspended Solids (VSS) concentration in the fermentation broth was determined according to standard methods [35]. Microbial biomass growth was monitored by measuring the absorbance of liquid samples at 600 nm using a spectrophotometer (DR2800, Hach Lange), and was correlated to the volatile suspended solids (VSS) concentration of the fermentation broth.

### Product yield and efficiency calculations

The product yields were calculated using CO, H_2_ and acetate (when applicable) as substrates and are given in mol product/e-mol substrate for them to be expressed on a common basis for all substrates according to:

1 $$Y_{i} \left( {\frac{\text{mol}}{\text{e-mol}}} \right) = \frac{{n_{(i)} {\text{produced}}}}{{n_{{{\text{H}}_{2} }} {\text{consumed}} \cdot n_{{e^{ - } {\text{H}}_{2} }} + n_{CO } \ {\text{consumed}} \cdot n_{{e^{ - } {\text{CO}}}} + n_{{{\text{Acetate}} }} \ {\text{consumed}} \cdot n_{{e^{ - } {\text{Acetate}}}} }}$$

where *n*_(*i*)_ is the number of moles of compound *i*, and $n_{{e^{ - } {\text{H}}_{2} }}$, $n_{{e^{ - } {\text{CO}}}}$ and $n_{{{\text{e-Acetate}} }}$ are the number of e-mol per mol of H_2_, CO and acetate, respectively. Acetate was included as substrate in the calculations only for enrichments and fermentations with initial addition of acetate that resulted in net consumption of acetate.

The efficiency of the enriched mixed microbial consortia was calculated based on the recovery of e-mol and carbon from the different substrates (H_2_, CO and CO_2_) in the products. The recovery of e-mol from H_2_ and CO in products was corrected by subtracting the total e-mol produced in control experiments according to:

2 $${\text{e-mol recovery}}\; (\% ) = \frac{{\mathop \sum \nolimits_{i = 1}^{N} \left( {n_{(i)} {\text{produced}} - n_{\left( i \right)} {\text{control}}} \right) \cdot n_{{e^{ - } \left( i \right)}} }}{{n_{{{\text{H}}_{2}}} {\text{consumed}} \cdot n_{{e^{ - } {\text{H}}_{2} }} + n_{{{\text{CO}}}}\ {\text{consumed}} \cdot n_{{e^{ - } {\text{CO}}}} }} \cdot 100$$

where $n_{{e^{ - } \left( i \right)}}$ is the number of e-mol per mol of compound *i*. Similarly, the recovery of carbon from CO and CO_2_ in the products was calculated taking into account the production in control experiments and the concentration of CO_2_ (aq), HCO_3_^−^, CO_3_^−2^ as a function of the initial and the final pH according to Eqs. 3, 4, 5 and 6.

3 $${\text{Cmol recovery}} \ (\% ) = \frac{{\mathop \sum \nolimits_{i = 1}^{N} {\text{Cmol}}_{(i)} {\text{produced}} - {\text{Cmol}}_{(i)} {\text{control}} }}{{n_{\text{CO}} {\text{consumed}} + n_{{{\text{CO}}_{2} }} {\text{consumed}} + n_{{{\text{HCO}}_{3}^{ - } }} {\text{consumed}} + n_{{{\text{CO}}_{3}^{ - 2} }} {\text{consumed}} }} \cdot 100$$

4 $$\left[ {{\text{CO}}_{2} ({\text{aq}})} \right] = K_{{{\text{H}} {\text{CO}}_{2} }} \cdot P_{{{\text{CO}}_{2} }}$$

5 $$\left[ {{\text{HCO}}_{3}^{ - } } \right] = \frac{{\left[ {{\text{CO}}_{2} ({\text{aq}})} \right] \cdot {\text{Ka}}_{1} }}{{\left[ {{\text{H}}^{ + } } \right]}}$$

6 $$\left[ {{\text{CO}}_{3}^{ - 2} } \right] = \frac{{{\text{Ka}}_{2} \cdot \left[ {{\text{CO}}_{2} \left( {\text{aq}} \right)} \right] \cdot {\text{Ka}}_{1} }}{{\left[ {{\text{H}}^{ + } } \right]^{2} }}$$

The dissociation constants Ka_1_ and Ka_2_ were corrected for the ionic strength of BA medium (*I* = 0.08 M) by solving Eq. 7 for Ka. The standard Gibbs free energy of formation (Δ_f_*G*°) of the carbonate species was extracted from Alberty [36] and corrected using the extended Debye–Hückel equation (Eq. 8) prior to calculating the Gibbs free energy change of the dissociation reactions (∆_r_*G*°) [37].

### Thermodynamic calculations

The thermodynamics of the net biochemical reactions was evaluated through the Gibbs free energy change (∆_r_*G*°) of reactions under the specific operating conditions of the enrichment series. To study the effect of the enrichment conditions on the feasibility of each biochemical reaction, the ∆_r_*G*° of all reactions was corrected for ionic strength, pH, temperature and concentration of substrates and products. Standard Gibbs free energies of formation (Δ_f_*G*°) and standard enthalpies of formation (Δ_f_*H*°) were extracted from Amend and Shock [38] and Alberty [36]. The Δ_f_*G*° and Δ_f_*H*° were corrected for the ionic strength of BA medium (*I* = 0.08 M) using the extended Debye–Hückel equation:

7 $$0 = \Delta_{r} G^\circ \left( {I = 0.08 M} \right) + RT\ln {\text{Ka}}$$

8 $$\Delta_{\text{f}} G^\circ_{i} \left( I \right) = \Delta_{\text{f}} G^\circ_{i} \left( {I = 0} \right) - \frac{{RTAz_{i}^{2} I^{1/2} }}{{1 + {\text{BI}}^{1/2} }}$$

9 $$\Delta_{\text{f}} H^\circ_{i} \left( I \right) = \Delta_{\text{f}} H^\circ_{i} \left( {I = 0} \right) + \frac{{1.4775z_{i}^{2} I^{1/2} }}{{1 + {\text{BI}}^{1/2} }}$$

where *z*_*i*_ is the charge number of compound *i*, A equals 1.1758 kg^1/2^/mol^1/2^ in water at 25 °C and B is an empirical constant that equals 1.6 L^1/2^/mol^1/2^ within a ionic strength range of 0.05–0.25 M [36]. Next, the initial concentration of products and substrates in the medium and partial pressure of gases was corrected using Eq. 10, and the pH was taken into account according to Steinbusch et al. [22]. Note that the reaction quotient in Eq. 10 denotes the concentration of products and reactants of the general reaction *aA* + *bB *↔ *cC* + *dD*. Finally, ∆_r_*G*′ of all reactions was adjusted to the incubation temperature of the enrichment experiments (310.15 K) using the Gibbs–Helmholtz Eq. 11. The reactions considered in the thermodynamic analysis are given in Table 2.

10 $$\Delta_{r} G^{\prime} = \Delta_{r} G^\circ \left( {I = 0.08 M} \right) + RT\ln \frac{{\left[ C \right]^{c} \left[ D \right]^{d} }}{{\left[ A \right]^{a} \left[ B \right]^{b} }}$$

11 $$\Delta_{r} G^{\prime}_{T} = \Delta_{r} G^{\prime}_{298.15 K} \cdot \frac{T}{298.15 K} + \Delta_{r} H^{\prime}_{298.15 K} \cdot \frac{298.15 K - T}{298.15 K}$$

**Table 2:** Biochemical reactions, ATP yield and average stoichiometric number used in the thermodynamic potential factor calculation

Stoichiometry of biochemical reactions	ATP yield (mol per reaction)	χ	Refs.
H_2_/CO_2_ conversion into acetate/ethanol			
4 H_2_ + 2 CO_2_ → CH_3_COO^−^ + H^+^ + 2 H_2_O	0.3	2	[40]
6 H_2_ + 2 CO_2_ → CH_3_CH_2_OH + 3 H_2_O	− 0.1/0.3^a^	3	[40]
CO conversion into acetate/ethanol			
4 CO + 2 H_2_O → CH_3_COO^−^ + H^+^ + 2 CO_2_	1.5	4	[40]
6 CO + 3 H_2_O → CH_3_CH_2_OH + 4 CO_2_	1.7	6	[40]
VFA reduction to corresponding alcohols			
CH_3_COO^−^ + H^+^ + 2 H_2_ → CH_3_CH_2_OH + H_2_O	0.33	4	[42]
CH_3_(CH_2_)_2_COO^−^ + H^+^ + 2 H_2_ → CH_3_(CH_2_)_2_CH_2_OH + H_2_O	0.33	4	[42]
CH_3_COO^−^ + H^+^ + 2 CO + H_2_O → CH_3_CH_2_OH + 2 CO_2_	0.66	5	
CH_3_(CH_2_)_2_COO^−^ + H^+^ + 2 CO + H_2_O → CH_3_(CH_2_)_2_CH_2_OH + 2 CO_2_	0.66	5	
Chain elongation			
5 CH_3_CH_2_OH + 3 CH_3_COO^−^ → 4 CH_3_(CH_2_)_2_COO^−^ + H^+^ + 3 H_2_O + 2 H_2_	1	1	[41]

^a^Bertsch and Müller [40] predicted an ATP yield of − 0.1; however, the possibility of a positive ATP yield was also evaluated

#### Thermodynamic potential factor (*F*_T_)

The thermodynamic potential factor (*F*_T_), introduced by Jin and Bethke [39], was calculated with the purpose of identifying a possible thermodynamic control on the rates of the biochemical reactions considered. Jin and Bethke [39] introduced this factor as a modification of usually employed rate laws, e.g., Monod equation ($r = \frac{{k_{ \text{max} } \cdot S}}{{k_{\text{s}} + S}} \cdot X \cdot F_{\text{T}}$), to make them thermodynamically consistent by taking into account the energy available and the energy conserved along a particular metabolic pathway. Thus, the thermodynamic potential factor (*F*_T_) determines whether the net biochemical reactions are subject to either thermodynamic or kinetic control depending on the thermodynamic driving force of each reaction, and is calculated according to the following equation:

12 $$F_{\text{T}} = 1 - { \exp }\left( { \frac{{\Delta {\text{G}}_{\text{A}} + \Delta {\text{G}}_{\text{C}} }}{\chi RT}} \right)$$

where ∆*G*_A_ corresponds to the energy available through each biochemical reaction (−∆_r_*G*′_310 K_ calculated as described in “Thermodynamic calculations”) in kJ per reaction; ∆*G*_C_ is the energy conserved determined by the number of ATP produced in each metabolic pathway multiplied by the Gibbs free energy of phosphorylation (∆*G*_p_); and χ is the average stoichiometric number and represents the number of times a rate-determining step takes place in the overall reaction or metabolic pathway. When ∆*G*_A_ ≫ ∆*G*_C_, there is a strong thermodynamic driving force for the forward reaction and *F*_T_ approaches 1, indicating that the rate of the reaction is strictly dependent on the kinetics of the biochemical reaction. Conversely, when ∆*G*_A_ approaches ∆*G*_C_, there is a small thermodynamic drive and *F*_T_ approaches 0, indicating a thermodynamic control of the reaction as *F*_T_ exerts a strong effect on the reaction rate. Finally, ∆*G*_A_ equal to or lower than ∆*G*_C_ results in a *F*_T_ of 0 or negative, respectively, indicating that the thermodynamic drive vanishes and the metabolism ceases.

The calculations of the *F*_T_ for the direct conversion of H_2_/CO_2_ and CO into acetate and ethanol were carried out using ATP yields given by Bertsch and Müller [40]. The chain elongation reaction was assumed to yield 1 mol ATP per reaction, obtained through substrate level phosphorylation, according to Angenent et al. [41]. The reduction of acetic acid into ethanol with H_2_ as electron donor was assumed to follow the acetate activation pathway via acetyl-CoA at the expense of 1 ATP, which would be compensated through the translocation of four protons across the membrane resulting in 0.33 mol ATP per reaction, as suggested by González-Cabaleiro et al. [42]. The analogous acetic acid reduction to ethanol using CO was assumed to follow the same pathway; however, a tentative yield of 0.66 mol of ATP per reaction was used, as the ATP yield of this reaction would be expected to be higher due to the oxidation of the additional reduced ferredoxin produced by CO dehydrogenase. The stoichiometry of the ATP synthesis through ion translocation used for the calculated ATP yields corresponded to a fixed ratio of 3 mol of H^+^/Na^+^ per mol of ATP. The average stoichiometric number (χ) was determined by the number of ions translocated across the membrane for all reactions, except for the chain elongation, in which the substrate level phosphorylation was used instead. It should be noted that the ATP yields used here were not determined experimentally, and thus, are subject to uncertainties derived from the assumptions made in each case. To account partially for these uncertainties and give an idea of the sensitivity of the thermodynamic potential factor (*F*_T_) to the energy conservation parameter, a rather broad range of Gibbs free energy of phosphorylation was used for the calculations corresponding to 45 kJ/mol ATP [43], 57.5 kJ/mol ATP and 70 kJ/mol ATP [44]. The values used for the calculations are summarized in Table 2.

## Results and discussion

### Enrichment of syngas-converting microbial consortia based on pH

#### Ethanologenic potential of enriched consortia

A number of enrichment strategies using the pH as the main selective driver were designed to study the evolution of the ethanologenic activity during the enrichment of microbial consortia at different initial conditions. Different initial pH conditions were tested using a heat-shock-treated inoculum (pH 6, 5.5 and 5) and a non-treated inoculum (pH 5).

All enrichment strategies successfully suppressed the methanogenic activity of the anaerobic sludge as methane was not detected in any transfer of the enrichments. This was expected in the enrichments using a heat-shock-treated inoculum, since spore-forming bacteria should be predominant in the anaerobic sludge as a result of the heat treatment [45]. In turn, small amounts of methane were expected when using the non-treated inoculum due to the abundance of methanogenic archaea in the anaerobic sludge, as observed by Steinbusch et al. [46]. However, in this study, the low initial pH (pH 5) of experiments using the non-treated inoculum inhibited both methanogenic and acetogenic growth when YE was not added to the growth medium, whereas experiments with YE addition presented exclusively acetogenic growth. This indicates that the methanogenic activity of the sludge was inhibited by the combination of low pH and toxicity of CO. Thus, open-mixed cultures could be used in syngas fermentation processes with no need of heat treatment or methanogenic inhibitors, just by operating at harsh conditions for methanogenic archaea.

**Fig. 2:**
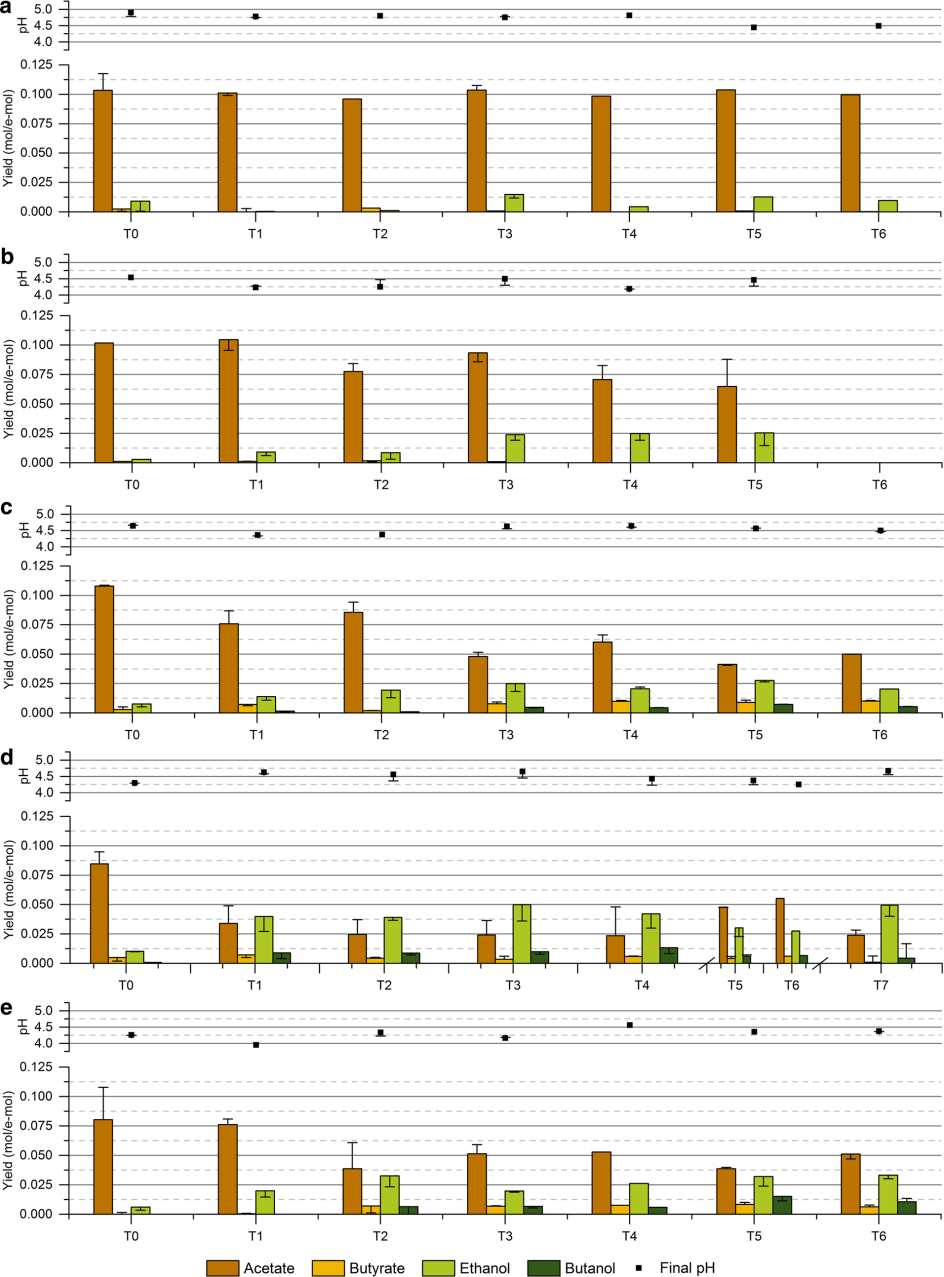
Product yields (mol/e-mol) obtained in each transfer for all enrichment conditions and final pH at the moment of the transfer. The columns show the values for the fermentation transferred and the error bars indicate the corresponding values of the duplicate experiment. Additional information on substrate consumption and apparent biomass yields can be found in Additional file 1: Figure S5. **a** Enrichment HT6 at an initial pH of 6; **b** enrichment HT5.5 at an initial pH of 5.5; **c** enrichment HT5.5YE at an initial pH of 5.5 with YE (0.5 g/l); **d** enrichment HT5YE at an initial pH of 5 with YE (0.5 g/l); **e** enrichment NT5YE at an initial pH of 5 with YE (0.5 g/l)

All enrichment conditions presented a very low ethanol production in the first transfer, with the product spectrum being initially dominated by acetate (Fig. 2). However, as the enrichments progressed, the distribution of products rapidly shifted following different trends depending on the operating conditions and seemed to be relatively stable after two to three transfers in most of the enrichments. The enrichment with initial pH 6 (HT6) resulted in the narrowest product spectrum with acetate as the main end product, and small amounts of ethanol and butyrate produced irregularly during the enrichment (Fig. 2a). As expected, decreasing the initial pH to 5.5 (HT5.5) improved the production of ethanol, which increased gradually along the enrichment reaching a maximum ethanol yield of 0.025 mol/e-mol (30.4% of the stoichiometric maximum) and an average of duplicates of 0.020 ± 0.005 mol/e-mol at transfer T5 (Fig. 2b). Nevertheless, the lower initial pH of this enrichment negatively affected the growth of the microbial consortium as the enrichment advanced until transfer T5, at which the culture could not be reactivated anymore. The enrichment with initial pH 5 (HT5) did not present any growth (data not shown); indicating that the microbial activity of the inoculum used was inhibited by the low initial pH of this enrichment. Consequently, enrichments with initial pH 5.5 and 5 were restarted and supplemented with YE to favor a better microbial growth. The addition of YE to the enrichment with initial pH 5.5 (HT5.5YE) indeed allowed a much better microbial growth as the lag phase of the culture was significantly reduced (Additional file 1: Figures S1–S4), and did not have any effect on the final ethanol yield when compared to enrichment HT5.5 (Fig. 2b, c). Further decreasing the initial pH to 5 significantly enhanced the solventogenic activity in enrichment HT5YE, with ethanol becoming the main end product and obtaining a maximum ethanol yield of 0.050 mol/e-mol (59.5% of the stoichiometric maximum) and an average of 0.045 ± 0.005 mol/e-mol at transfer T7 (Fig. 2d).

The enrichment with initial pH 5 using the non-treated inoculum (NT5YE) presented a similar trend with enrichment HT5YE, as the solventogenic activity was rapidly boosted along the successive transfers (Fig. 2e). However, the maximum ethanol yield obtained in enrichment NT5YE at transfer T6, namely, 0.033 mol/e-mol and 39.7% of the stoichiometric maximum (average of 0.032 ± 0.002 mol/e-mol), was not as high as that of enrichment HT5YE. An explanation for this difference in the product yields could be based on the heat treatment of the inoculum, as the enrichment HT5YE presented a more abrupt response upon exposure to the enrichment conditions (from T0 to T1) compared to enrichment NT5YE, where changes in the product profile took place gradually (from T0 to T2). Thus, it is possible that the higher degree of sporulation derived from the heat treatment of the initial inoculum favored a faster microbial selection process ultimately resulting in a different ethanologenic activity in these two enriched consortia. Besides the quantitative difference in the final ethanol yield, the high similarity in the behavioral traits of these microbial consortia is also supported by their common response upon reactivation of the cultures. As shown in Fig. 2d, e, a noticeable decrease of solventogenic activity was observed in both enrichments after stopping them for 2 months at transfer T4 (HT5YE) and T2 (NT5YE). Additionally, a very similar or even higher solventogenic activity was recovered after two transfers upon resuming the enrichments in both microbial consortia.

The apparent biomass yield was observed to be affected by both the initial pH of the enrichments and the addition of YE. Generally, the average biomass yield along the enrichments varied between 1.7 and 2.8 mg VSS/e-mol, with enrichment HT5.5 presenting the lowest biomass yield and enrichments HT5YE and NT5YE exhibiting the highest biomass yields (Additional file 1: Figure S5). No statistically significant differences were found between the average biomass yield of the enrichments at different pH, with *P* values above 0.05 in all cases when comparing HT6 to the rest of the enrichments (Additional file 1: Figure S5 and Table S1). However, the fact that the enrichment HT5.5 could not be reactivated at transfer T6 and that enrichment HT5 did not present any growth indicates a clear negative effect of the pH on microbial growth. In turn, the addition of YE was observed to improve the biomass yield of the enrichment cultures as statistically significant differences with *P* values below 0.05 were found between enrichment HT5.5 and all enrichments with YE addition, namely, HT5.5YE, HT5YE and NT5YE (Additional file 1: Table S1).

A low pH is commonly applied in syngas fermentation studies [47, 48] based on the hypothesis that the higher diffusion of VFAs through the cell membrane at acidic pH triggers solventogenesis as a means of preventing a further intracellular pH drop [49, 50]. The observations made in this study are in agreement with this hypothesis as (i) the highest ethanol yields were obtained in the enrichments at the lowest initial pH tested, and (ii) the final pH of the fermentations oscillated around 4.3–4.6 in most enrichment conditions and seemed not to be related to the initial pH conditions (Fig. 2c, d). Thus, it is likely that intracellular pH homeostasis may have driven a higher ethanol production by the enriched consortia.

The addition of YE appeared to have no effect on the final yield of ethanol in enrichments at an initial pH of 5.5 (Fig. 2b, c), but triggered the production of butyrate, butanol and small amounts of caproate leading to a broader product spectrum in all YE-supplemented enrichments (Fig. 2c–e). The production of butyrate and butanol was observed to take place in a two-step reaction, which indicates that they were produced through chain elongation and reduction of VFAs (Additional file 1: Figures S6, S7). The potential of microbial consortia for producing medium-chain fatty acids (MCFAs) through chain elongation has been shown in a number of studies [18, 51]. However, when it comes to ethanol production, the chain elongation process is often regarded as a major drawback as it reduces the selectivity of the mixed culture towards ethanol due to the conversion of ethanol and VFAs into MCFAs, as found in El-gammal et al. [52]. In this study, a significant chain-elongating activity appeared to be prevented by the low pH of the fermentations (generally ranging between 4.3 and 4.6 at the end of the experiments), since both acetate and ethanol remained as the major end products at all enrichment conditions. Ganigué et al. [21] reached similar conclusions in a study targeting the production of higher alcohols, in which low pH was found to affect negatively the chain elongation process. Nevertheless, the reduced chain-elongating activity found in the present study allowed achieving high ethanol yield in enrichments at pH 5.

A maximum ethanol yield of 0.050 mol/e-mol (59.5% of e-mol recovery) and an ethanol-to-acetate ratio of 1.58 g/g was achieved in enrichment HT5YE. The maximum ethanol-to-acetate ratio obtained was significantly higher than those often reported in other batch experiments using pure cultures such as *C. ragsdalei* (1.30 g/g) [10], *C. autoethanogenum* (0.39 g/g) [8] and *C. ljungdahlii* (0.70 g/g) [53], and in other mixed-culture studies with ratios below 0.4 g/g [20, 52]. Yet, higher ethanol-to-acetate ratios were reported by Singla et al. (2014) using the enriched culture TERI-SA1 (2.46 g/g).

The analysis of the production efficiency on fermentations carried out by the enriched consortia showed that both the pH and YE had a significant effect on the performance of the cultures. Table 3 shows the production efficiencies, calculated on a Cmol and e-mol recovery basis, for all enriched consortia. The calculated Cmol and e-mol recoveries were in relatively good agreement, with a maximum deviation of 10% corresponding to the enriched consortium HT6. Generally, the efficiency of the fermentations remained at a high levels for all enriched consortia and was consistent with previously reported e-mol recoveries [54]. Nonetheless, differences can be observed in Table 3, where the production efficiency of the enriched consortium HT5.5 resulted to be much lower than that of the consortia HT6 and HT5.5YE. A statistically significant difference was found when comparing the Cmol recovery of the enriched cultures HT5.5 and HT5.5YE (*P* value of 0.0003), indicating that the addition of YE had a positive effect on the production efficiency. On the other hand, the decrease in pH had an adverse effect on the production efficiency since a statistically significant difference (*P* value of 0.017 and 0.012 for Cmol and e-mol recovery, respectively) was found between the product recovery of the consortia enriched at pH 5.5 (HT5.5) and at pH 6 (HT6). However, the negative effects of reducing the initial pH were not significant as long as YE was added to the medium (Additional file 1: Table S2). These effects of pH and YE on the production efficiency were in fact anticipated as increasing both pH (in the range tested) and YE has been previously reported to favor biomass growth [8], which in turn reduces the maintenance requirements for cell metabolism and allows a higher production efficiency. Interestingly, the product distribution had no effect on the production efficiency of the fermentations, indicating that the latter was strictly dependent on the growth conditions.

**Table 3:** Efficiency calculated in terms of e-mol and Cmol recovery and product yields for all enriched microbial consortia

	HT6	HT5.5^a^	HT5.5YE	HT5YE	NT5YE
Recovery (%)^b^					
e-mol	92.92 ± 0.54	85.83 ± 2.46	89.84 ± 1.80	88.57 ± 1.77	95.01 ± 3.29
Cmol	83.16 ± 0.70	77.33 ± 2.31	93.18 ± 1.69	80.29 ± 0.83	91.60 ± 6.41
Product yield (% e-mol/e-mol)					
Acetate	83.32 ± 0.81	61.15 ± 7.41	41.38 ± 1.75	29.29 ± 0.63	27.68 ± 1.67
Propionate	0.00 ± 0.00	0.11 ± 0.20	0.01 ± 0.08	2.11 ± 0.38	0.26 ± 0.04
Iso-butyrate	1.55 ± 0.09	0.00 ± 0.00	1.82 ± 0.78	0.00 ± 0.00	1.17 ± 0.31
Butyrate	0.00 ± 0.00	0.00 ± 0.00	11.59 ± 4.82	2.05 ± 0.74	16.51 ± 1.07
Ethanol	8.16 ± 0.49	25.20 ± 5.32	29.40 ± 5.36	59.15 ± 0.18	34.81 ± 2.27
Butanol	0.00 ± 0.00	0.00 ± 0.00	15.37 ± 1.55	3.50 ± 0.64	21.82 ± 1.45
Caproate	0.00 ± 0.00	0.00 ± 0.00	1.39 ± 0.03	3.33 ± 0.20	1.09 ± 0.09
Biomass yield (g VSS/e-mol)	2.10 ± 0.11	1.83 ± 0.42	3.21 ± 0.51	3.13 ± 0.20	2.85 ± 1.06

^a^The efficiency and product yields of HT5.5 were calculated using the results for transfers T4 and T5 of the enrichment

^b^Additional information on the production of control experiments is provided in Additional file 1: Table S3

#### Microbial characterization of enrichment cultures

A total of 49,736,621 sequences were obtained from all investigated samples after quality checking and data filtering, with an average of 956,473 reads per sample (range 309.337–8,985,816 reads per sample). Replication, error correction, denoising with unoise algorithm and filtering of OTU table resulted in 6183 OTUs with all but one OTUs belonging to bacteria domain. Considering all sequences retrieved in the present study, Firmicutes accounted for the largest fraction (78% of the total), mainly represented by the classes *Clostridia*, *Tissierella* and *Bacilli*.

**Fig. 3:**
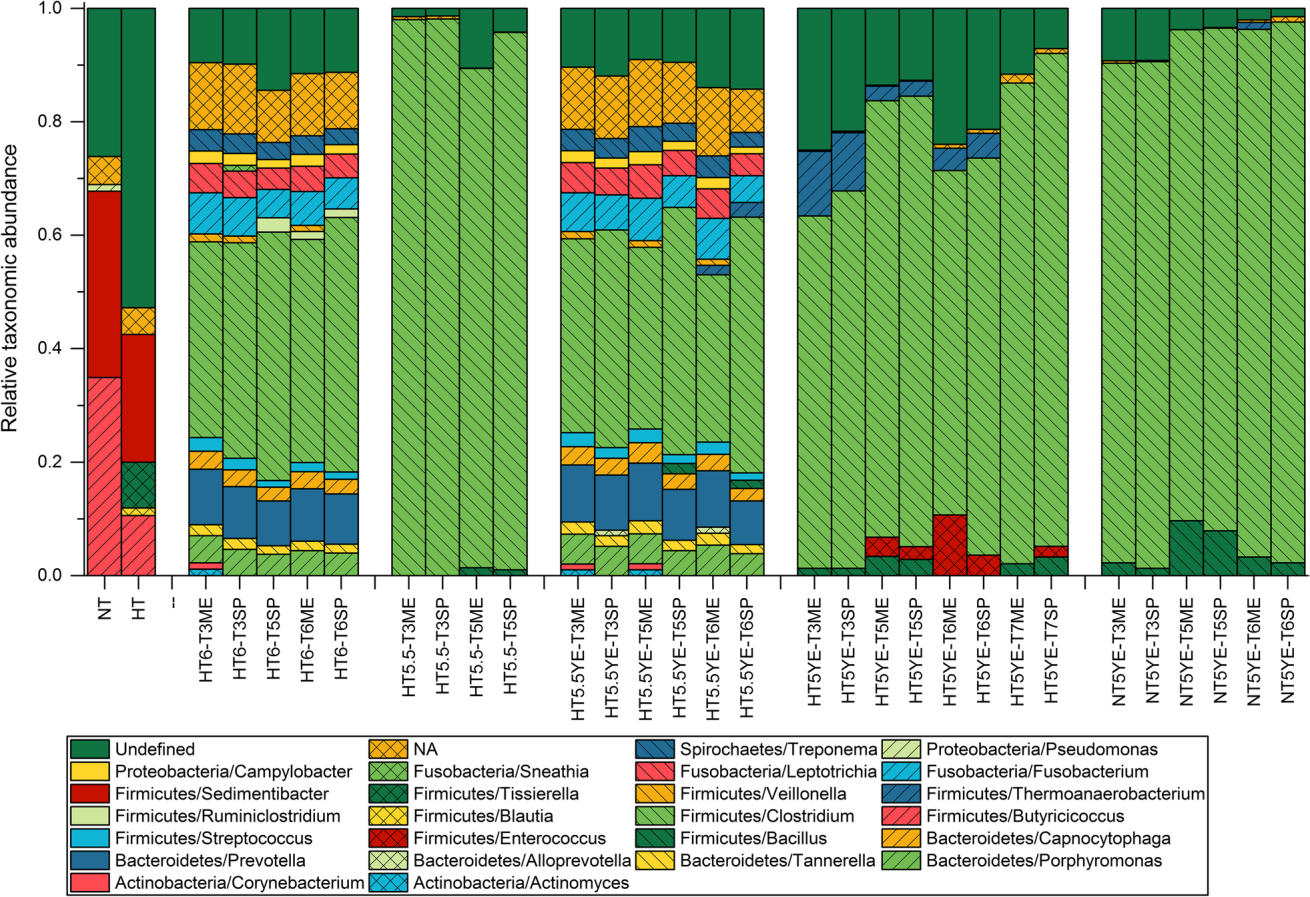
Relative taxonomic abundance of the analyzed microbial consortia in pH-based enrichments at genus level. The label of the samples is encoded according to the enrichment name, transfer number and growth phase at the moment of the sampling. ME and SP stand for mid-exponential and stationary growth phase, respectively. The label NA corresponds to the sum of all genera with a relative abundance below 1%

The analysis of the microbial composition of the enrichment samples at genus level revealed significant differences between the initial inocula and the different enrichment cultures. The untreated (NT) and heat-shock-treated (HT) inocula were initially dominated by the genera *Sedimentibacter* (32.8% and 22.5% of reads mapping to corresponding OTUs for NT and HT, respectively) and *Butyricicoccus* (34.9 and 10.6% for NT and HT, respectively), while other genera like *Clostridium* represented only a minor fraction (below 1% in both cases) (Additional file 2: Table S1). However, upon exposure to the different operating conditions, the composition of all enrichment cultures rapidly shifted with a clear and stable dominance of the genus *Clostridium* from transfer T3. As shown in Fig. 3, the degree of dominance of the genus *Clostridium* was variable across the different enrichment conditions. Samples from enrichments HT6 and HT5.5YE presented the highest diversity in terms of genera, with the genus *Clostridium* representing an average of 41.5 ± 3.3% and 37.1 ± 6.3% of the total reads mapped, respectively. In turn, samples from enrichments HT5.5, HT5YE and NT5YE exhibited a lower diversity at genus level with a much larger representation of the reads mapping to OTUs corresponding to *Clostridium* with an average of 94.7 ± 4.7%, 73.4 ± 10.0% and 90.2 ± 3.3%, respectively. These results show that *Clostridium* was among the most resilient genera found in the microbial consortia since its dominance increased with harsher enrichment conditions, i.e., decreasing pH or absence of YE.

Despite the clear dominance of OTUs belonging to the genus *Clostridium* in all enrichments, the abundance of individual OTUs found in the enrichment samples varied depending on the enrichment conditions (Additional file 2: Table S3). The mean relative frequencies between the OTU abundances were compared across all samples with and without addition of YE. It was found that representative sequences of abundant OTUs identified solely in enrichments without addition of YE (HT6 and HT5.5), aligned with high identity (98.4–100%) to 16S rRNA genes from *Clostridium autoethanogenum* and *Clostridium ljungdahlii.* Abundances of these OTUs (2;1302;1233;1249;1983) were 81.5% and 41.4% in samples HT5.5T5SP and HT6T6SP, respectively (Additional file 2: Table S3). In turn, reads mapping to these OTUs were negligible in YE-supplemented enrichments. Moreover, identified OTUs exclusively present in YE-supplemented enrichments and their representative sequences exhibited the best alignment (97–100% identity) with 16S rRNA sequences of *Clostridium drakei* and C*lostridium carboxidivorans.* Abundance of these OTUs (1;337;434;359) was up to 87.5% in sample NT5YET6SP (Additional file 2: Table S3). These differences indicate that the addition of YE, besides promoting better growth conditions for the entire microbial consortium, also played a determining role as a selection factor in addition to the pH conditions and the substrate composition.

The range of metabolites found in each of the enrichment series was in agreement with the product portfolio of the putative dominant species identified in the enrichment samples. As mentioned above, acetate and ethanol were the main metabolites in enrichments HT6 and HT5.5 where the putative dominant species were *C. autoethanogenum* and *C. ljungdahlii*, while longer carbon chain products such as butyrate and butanol were also found in YE-supplemented enrichments (HT5.5YE, HT5YE and NT5YE) with *C. drakei* and *C. carboxidivorans* as putative dominant species. Both *C. autoethanogenum* and *C. ljungdahlii* have been reported to produce only acetate and ethanol when fermenting syngas in batch cultures [55, 56]. In turn, *C. drakei* and *C. carboxidivorans* present a broader product spectrum including butyrate, butanol, and even caproate and hexanol in the case of *C. carboxidivorans* [57, 58], which are produced through re-assimilation, chain elongation and reduction of the primary metabolites [59]. Additionally, the optimum growth conditions for all these species vary between a pH of 5.5 and 6.2 and temperatures around 37 °C, which explains the dominance of these species during the enrichments. Therefore, most likely the dominant species identified were the major contributors to the formation of products observed during the enrichments.

The ethanol yield seemed to be independent of the microbial composition of the enrichment cultures at genus level. Enrichments HT6 and HT5.5YE resulted in a similar microbial composition (Fig. 3) and presented a maximum ethanol yield along the enrichment of 0.015 and 0.028 mol/e-mol, respectively (Fig. 2). Similarly, enrichments HT5.5, HT5YE and NT5YE also presented similar microbial composition with a clear dominance of the genus *Clostridium* (Fig. 3) and resulted in a maximum ethanol yield of 0.025 mol/e-mol, 0.050 mol/e-mol and 0.034 mol/e-mol, respectively (Fig. 2). On the contrary, enrichments at similar operating conditions and different microbial composition (HT5.5 and HT5.5YE) resulted in similar maximum ethanol yields. Thus, despite the clear effect of the pH on the microbial composition of the enriched consortia, it can be concluded that the shift towards ethanol observed in the enrichment experiments was probably the result of the metabolic response to the different initial pH conditions and not so dependent on the microbial composition of the enrichment cultures.

A direct comparison with the literature is not possible since the only quantitative analysis of the composition of a syngas-converting microbial consortium using a 16S rRNA amplicon-based sequencing method was performed under higher pH conditions (pH 7.5), and thus, resulted in significantly different microbial composition [54]. However, the species identified in other studies carried out at a comparable pH range (pH 6.2–6.0) which were entirely consistent with the results found here [21, 24]. Ganigué et al. (2016) studied the composition of a microbial consortium during the conversion of syngas into higher alcohols using PCR-DGGE analysis and identified both of the putative dominant species reported here (*C. autoethanogenum*/*C. ljungdahlii* and *C. drakei*/*C. carboxidivorans*). In their study, *C. autoethanogenum/C. ljungdahlii* were found to be the main species carrying out the carbon fixation. In turn, Singla et al. [60] found that *C. drakei* and *C. scatalogenes* were either the main or possibly the only members of the enriched microbial consortium TERI-SA1. Interestingly, YE was not added in the medium used by Ganigué et al. [21] during the enrichment, while Singla et al. [60] added 1 g/l of YE to the medium. Taking this into consideration, the findings reported in the literature and the results reported here follow the same trend, with the putative dominant species of the enrichment cultures being *C. autoethanogenum*/*C. ljungdahlii* when YE was absent in the growth medium and *C. drakei*/*C. carboxidivorans* when YE was added to the medium.

#### Thermodynamic analysis of the metabolic network of the enriched consortia

The enriched consortia developed through pH-based enrichments were observed to produce ethanol with relatively high selectivity. However, the production of ethanol took place during the exponential phase of the fermentations, and thus, it was not possible to distinguish between direct production of ethanol and reduction of acetic acid to ethanol. Therefore, a thermodynamic analysis of the metabolic network of net biochemical reactions taking place during the activity of the enriched consortia was performed to identify possible bioenergetic drivers of the metabolic shift observed under different enrichment conditions. Based on experimental observations, the reactions considered for evaluating the ∆_r_*G*′_310 K_ and the thermodynamic potential factor (*F*_T_) under changing process conditions were the production of ethanol and acetic acid from H_2_/CO_2_ and CO, the production of butyric acid through chain elongation, and the reduction of both acetic and butyric acid into their corresponding alcohols using either H_2_ or CO as electron donor.

**Fig. 4:**
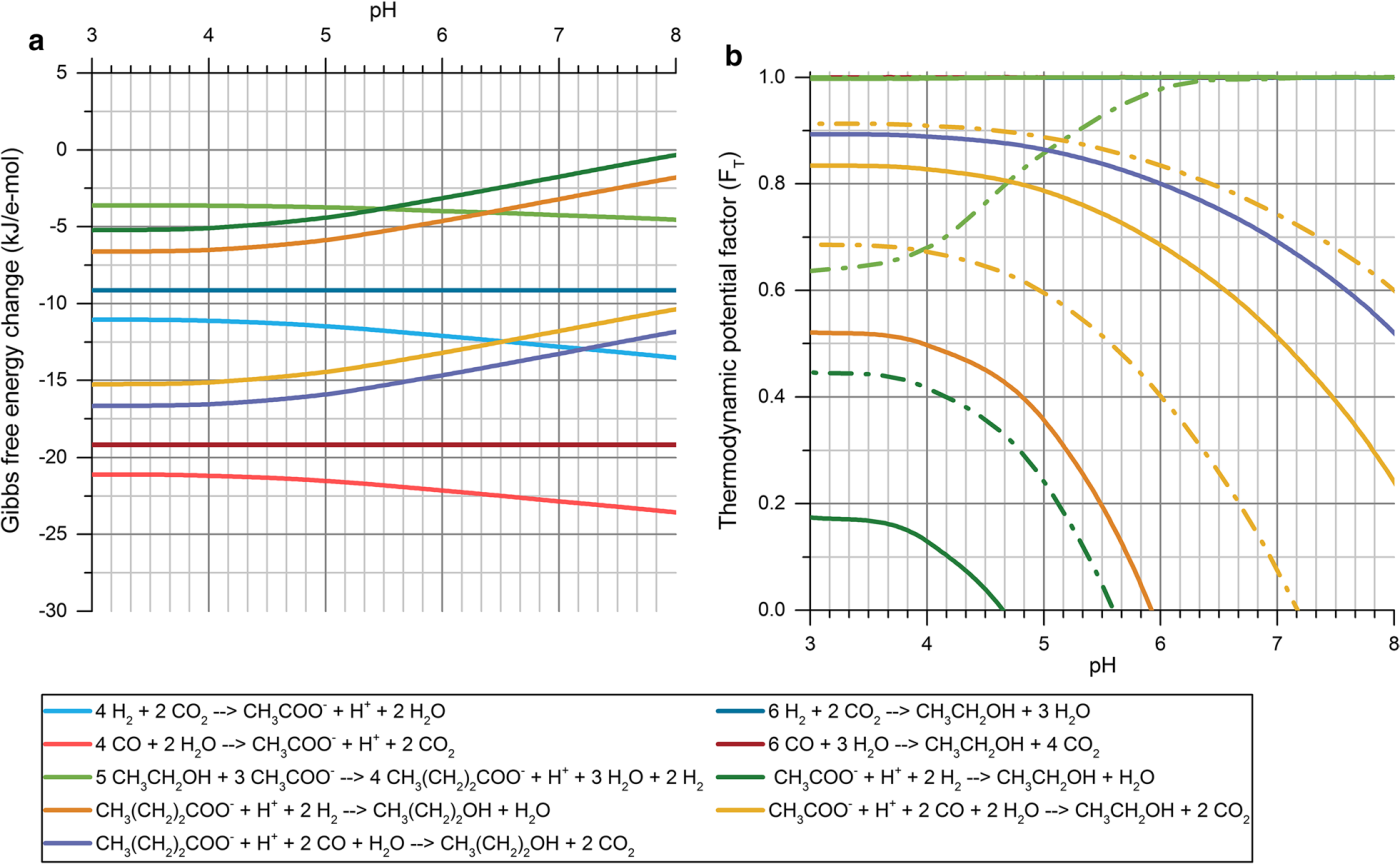
**a** Calculated ∆_r_*G*′_310 K_ for the metabolic network of microbial consortia as a function of pH, normalized for e-mol transferred per reaction. Process conditions considered were: temperature of 310.15 K, ionic strength of 0.08 M, P_H2_ of 1.05 atm, P_CO2_ of 0.6 atm, P_CO_ of 0.45 atm and concentration of metabolites of 0.001 M. **b** Thermodynamic potential factor calculated for all reactions as a function of pH. Solid lines represent *F*_T_ calculated using a ∆*G*_p_ of 57.5 kJ/mol of ATP. Dashed lines represent the upper and lower boundary of *F*_T_ when using a ∆*G*_p_ of 45 and 70 kJ/mol of ATP, respectively. The upper and lower boundaries are shown only for acetate-reducing reactions and the chain-elongation reaction

Analyzing the ∆_r_*G*′_310 K_ of the metabolic network of the enriched microbial consortia revealed that several reactions could be affected upon changing the initial pH conditions. As shown in Fig. 4a, the production of acetate would be favored over ethanol when considering the direct conversion of either H_2_/CO_2_ or CO, as the ∆_r_*G*′_310 K_ of acetate-producing reactions are always below that of ethanol regardless the pH conditions. However, acetate-producing reactions from both substrates become less exergonic as the pH decreases, while the analogous ethanol-producing reactions remain unaffected by pH changes. The chain elongation to butyrate follows a similar trend with acetate production, becoming thermodynamically less favorable upon decreasing the pH. In turn, the reduction of acetic and butyric acid using either H_2_ or CO as electron donor is significantly boosted by the pH decrease until the ∆_r_*G*′_310 K_ stabilizes at around pH 3.5–4. Overall, besides the fact that acid-producing reactions are negatively affected by the lower pH, it seems that the only reactions clearly favored upon decreasing the initial pH conditions are the reduction of VFAs into their corresponding alcohols. However, the analysis of the thermodynamic potential factor (*F*_T_) shows that not all reactions are equally affected by the changes in ∆_r_*G*′_310 K_ liberated. The *F*_T_ of both acetate- and ethanol-producing reactions from H_2_/CO_2_ and CO approach 1 at all pH conditions, indicating that these reactions would provide enough thermodynamic driving force to proceed forward regardless of the pH conditions considered and would not be significantly affected by the changes in ∆_r_*G*′_310 K_ (Fig. 4). Therefore, rather than being thermodynamically controlled, the rates of direct production of either acetate or ethanol would be ultimately controlled by the specific enzyme kinetics of each metabolic pathway, which in turn would be dependent on the concentration of intermediate metabolites and reduced cofactors during the fermentation. On the other hand, the evaluation of the *F*_T_ for the reduction of VFAs into their corresponding alcohols, using either H_2_ or CO, and the chain elongation reaction resulted in values between 0 and 1 depending on the pH conditions considered (Fig. 4b). This implies that the energy generated through these reactions is close to their energy conservation requirements, and as a result, the thermodynamic drive for these reactions to proceed forward is limited and pH-dependent. As opposed to direct acetate- and ethanol-producing reactions, the feasibility and the rate of VFA-reducing and chain elongation reactions are very sensitive to the changes in ∆_r_*G*′_310 K_ obtained at different pH conditions (Fig. 4a).

According to the thermodynamic analysis, the direct conversion of H_2_/CO_2_ and CO into either acetate or ethanol is not expected to be thermodynamically controlled until these substrates become severely depleted. However, the higher thermodynamic driving force (lower ∆_r_*G*′_310 K_) and ATP yield per mol of substrate for acetate-producing reactions suggest that these would prevail over direct ethanol-producing reactions under kinetic control. Besides, calculations carried out by Bertsch and Müller [40] for the model organism *Acetobacterium woodii* indicate that the production of ethanol from H_2_/CO_2_ might not be possible, as this reaction would require a net input of 0.1 mol of ATP per mol of ethanol.

As opposed to direct conversion route from H_2_/CO_2_ and CO to liquid products, VFA-reducing reactions are subject to thermodynamic control under the operating conditions considered and are clearly favored upon decreasing the pH. The *F*_T_ values for all VFA-reducing reactions show that changes in ∆_r_*G*′_310 K_ at the pH range studied have a strong effect on the rates of these reactions, which could explain the higher ethanol yield obtained in the enrichment experiments at an initial pH of 5. As illustrated in Fig. 4b, a high pH in the fermentation broth renders the reduction of acetate with H_2_ unfeasible (negative *F*_T_ values). However, the boundaries of feasibility for this reaction cannot be accurately delimited due to the high sensitivity of *F*_T_ to the values of ATP yield and Gibbs free energy of phosphorylation (∆*G*_p_) used in the calculations. Considering an ATP yield of 0.33 mol per reaction and a ∆*G*_p_ of 45 kJ/mol of ATP, the reduction of acetate would be feasible below a pH of 5.6, whereas using a ∆*G*_p_ of 70 kJ/mol of ATP would render this reaction unfeasible at any pH resulting in a maximum *F*_T_ of − 0.23 at pH 3. Thus, detailed conclusions on whether this reaction is possible as a function of pH cannot be drawn, although it is obvious that this reaction is more likely to occur at the lower range of pH studied. On the other hand, using CO as electron donor for the reduction of acetate provides a much lower ∆_r_*G*′_310 K_, reducing the uncertainties on the activity of this reaction. In this case, the use of CO as electron donor is possible at all conditions regardless of the ∆*G*_p_ considered (Fig. 4b). Furthermore, decreasing the pH from 6 to 5 causes the *F*_T_ of this reaction to increase from 0.68 to 0.79 (Fig. 4b), indicating that the acetate-reducing activity is significantly boosted as the pH decreases. Thus, it can be concluded that acetate-reducing reactions played an important role on the solventogenic activity observed in the enrichment experiments. Additionally, based on the *F*_T_ for these reactions, the acetate-reducing activity using CO rather than H_2_ as electron donor was probably more significant during the enrichments. This is in line with the observations made by Hu et al. [61] while studying the thermodynamics of the oxidation of CO and H_2_, where it was concluded that the use of CO as a source of electrons is thermodynamically more favorable than H_2_ at all conditions.

In “Ethanologenic potential of enriched consortia”, it was hypothesized that the chain elongation reaction was inhibited by the decrease of pH during the fermentation, as both acetate and ethanol remained as the main products of the fermentation and were only partially converted into butyrate (Additional file 1: Figures S6, S7). However, the results of the *F*_T_ for this reaction at different pH conditions show that the chain elongating activity is negatively affected by the decrease of pH when considering a ∆*G*_p_ of 70 kJ/mol, with *F*_T_ values corresponding to 0.98, 0.86 and 0.68 at pH 6, 5 and 4, respectively (Fig. 4b). Therefore, the inhibition due to pH drop observed experimentally could be grounded on a limitation in the thermodynamic driving force for this reaction to proceed forward.

The thermodynamic analysis carried out here suggests that a significant amount of ethanol was produced via a two-step reaction, where direct production of acetic acid was initially favored followed by its reduction into ethanol in a second step. This is consistent with the distinction between acidogenic and solventogenic growth phases commonly applied in syngas fermentation processes [48, 62]. Furthermore, the limited thermodynamic drive for chain-elongating activity found when decreasing the pH could explain the high selectivity towards ethanol observed in enrichments at pH 5. Thus, the methodological approach used here proved to be useful for a qualitative interpretation of how the metabolic network of mixed microbial consortia responds when changing operational conditions. Of course, microbial growth inhibition phenomena due to high VFAs/solvents concentration are not taken into account in this method and need to be considered from a microbiological perspective; however, the enrichment experiments took place at low substrate and product concentration and were not expected to present such inhibition phenomena. This method presented low accuracy when attempting to draw definite boundaries on the feasibility of the acetate reduction with H_2_ due to the broad range of ∆*G*_p_ used in the calculations. Other limitations identified were the fact that the energy conservation requirements, determined by the ATP yield and the ∆*G*_p_, were assumed to be constant regardless of the reaction and operating conditions considered. The stoichiometry of ATP synthesis was also assumed to have a fixed ratio of 3 ions translocated per mol of ATP. Nevertheless, both the ∆*G*_p_ and the stoichiometry of ATP synthesis have been shown to be subject to variation depending on several factors such as intracellular ATP/ADP ratio, electrochemical membrane potential, electron donors and acceptors considered, or even the species carrying out the reaction [44].

Despite the limitations outlined above, the thermodynamic analysis allowed for interpretation of the effects of operating conditions on the network of biochemical reactions prevailing in mixed microbial consortia. Thus, this method could be used for the selection of operational conditions with the aim of boosting specific reactions. To test the validity of this method for predicting changes in the microbial activity of enriched consortia and improving further the ethanol yield obtained previously, an additional experiment series was performed.

### Enrichment strategies based on thermodynamics of the metabolic network

#### Thermodynamic predictions of the microbial activity

From a thermodynamic perspective, the metabolic network of microbial consortia can be affected by several operating parameters such as the partial pressure of gases, the concentration of products or the pH. Several of these parameters could potentially enhance the production of ethanol due to their distinct effect on different reactions such as the pH already discussed, the partial pressure of CO_2_ given the distinct stoichiometric CO_2_ formation in acetate- and ethanol-producing reactions, the partial pressure of H_2_ and CO affecting acetate-reducing reactions, and the initial concentration of acetate and ethanol affecting the whole metabolic network. In this case, given the effect of the pH on the acetate-reducing activity discussed in “Thermodynamic analysis of the metabolic network of the enriched consortia”, it was decided to study the effect of the initial acetate concentration in the medium to boost these reactions even further. However, this can be regarded as a proof-of-concept since this method could be used to evaluate the effect of the abovementioned parameters on any reactions taking place under thermodynamic control, e.g., in systems operating in continuous mode under substrate-limiting conditions.

**Fig. 5:**
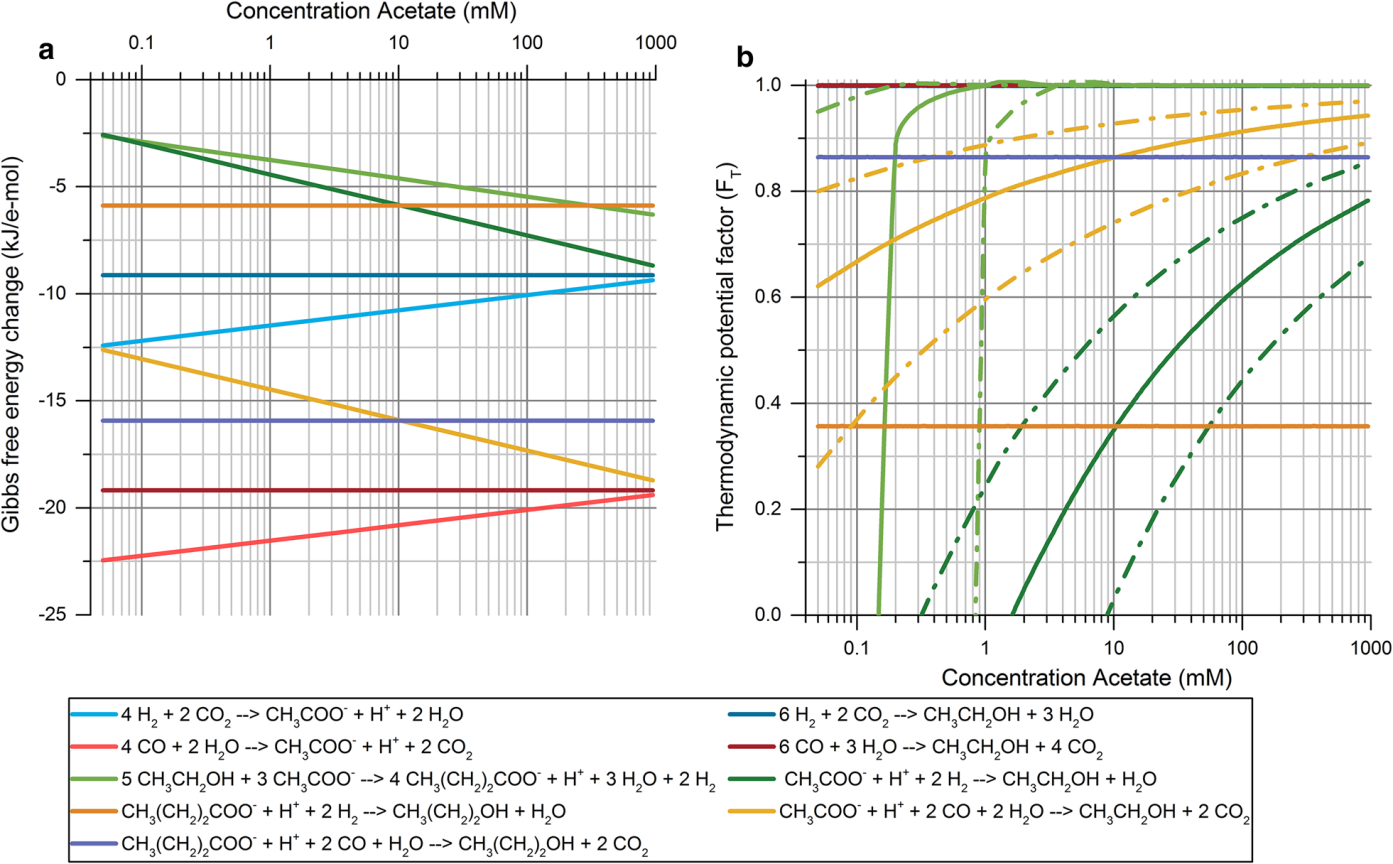
**a** Calculated ∆r*G*′_310 K_ for the metabolic network of microbial consortia as a function of acetate concentration, normalized for e-mol transferred per reaction. Process conditions considered were: temperature of 310.15 K, ionic strength of 0.08 M, P_H2_ of 1.05 atm, P_CO2_ of 0.6 atm, P_CO_ of 0.45 atm, concentration of other metabolites of 0.001 M and pH 5. **b** Thermodynamic potential factor calculated for all reactions as a function of pH. Solid lines represent *F*_T_ calculated using a ∆*G*_p_ of 57.5 kJ/mol of ATP. Dashed lines represent the upper and lower boundary of F_T_ when using a ∆*G*_p_ of 45 and 70 kJ/mol of ATP, respectively. The upper and lower boundaries are shown only for acetate-reducing reactions and the chain elongation reaction

The analysis of the ∆_r_*G*′_310 K_ indicated that the results obtained at pH 5 could be further improved by changing the initial concentration of acetate as several reactions would be significantly affected. As shown in Fig. 5a, the ∆_r_*G′*_310 K_ of ethanol-producing reactions from H_2_/CO_2_ and CO would be neither positively nor negatively affected by the initial concentration of acetate. In turn, all reactions involving the use of acetate as product or substrate present significant variations in the ∆_r_*G′*_310 K_ upon changes in acetate concentration. While acetate-producing reactions are negatively affected by the increase of acetate, the reactions consuming acetate become thermodynamically favored. However, based on the *F*_T_ obtained for each reaction, only acetate-reducing reactions and the chain elongation could be thermodynamically controlled upon changing the initial acetate concentration (Fig. 5b). The production of acetate from both H_2_/CO_2_ and CO would not be sensitive to changes in ∆_r_*G*′_310 K_, as the free energy liberated would be high enough to drive these reactions forward independently of the concentration of acetate (Fig. 5b).

Comparing the effect of the pH and the initial acetate concentration on acetate-reducing reactions, the analysis of the changes in ∆_r_*G*′_310 K_ and *F*_T_ shows that the concentration of acetate has a stronger effect on the activity rates of these reactions. An increase in acetate concentration from 1 mM to 20 mM would significantly boost the acetate-reducing activity as the *F*_T_ would increase from 0.79 to 0.88 when using CO as electron donor, and from − 0.13 to 0.45 when using H_2_. In this case, at an initial acetate concentration of 20 mM, both reactions would be clearly feasible regardless of the ∆*G*_p_ considered, even when using the more conservative ∆*G*_p_ of 70 kJ/mol of ATP (Fig. 5b). Thus, according to these calculations, an enrichment at pH 5 and 20 mM of initial concentration of acetate would be expected to boost the ethanologenic potential of the microbial consortium by increasing its acetate-reducing activity.

As opposed to lowering the pH, increasing the initial concentration of acetate would favor the chain-elongating activity. In this case, the F_T_ for chain elongation would remain constant at values approaching 1 by increasing the initial concentration of acetate from 1 mM to 20 mM when using a ∆*G*_p_ of 57.5 kJ/mol of ATP (Fig. 5b). Considering the most conservative ∆*G*_p_ of 70 kJ/mol of ATP, the *F*_T_ would increase from 0.86 to approx. 1. Therefore, the rate of this reaction would be clearly boosted by the increase of initial acetate concentration in the fermentation broth. This would theoretically decrease the ethanologenic potential of the microbial consortium, as also shown experimentally by El-gammal et al. [52]. However, as the pH was anticipated to decrease during the course of the fermentation, the inhibition of the chain-elongating activity due to the low pH observed in this and other studies [21] was expected to play an important role in such enrichment conditions by preventing a significant activity.

#### Enrichment with acetate addition: ethanol yield and microbial community

**Fig. 6:**
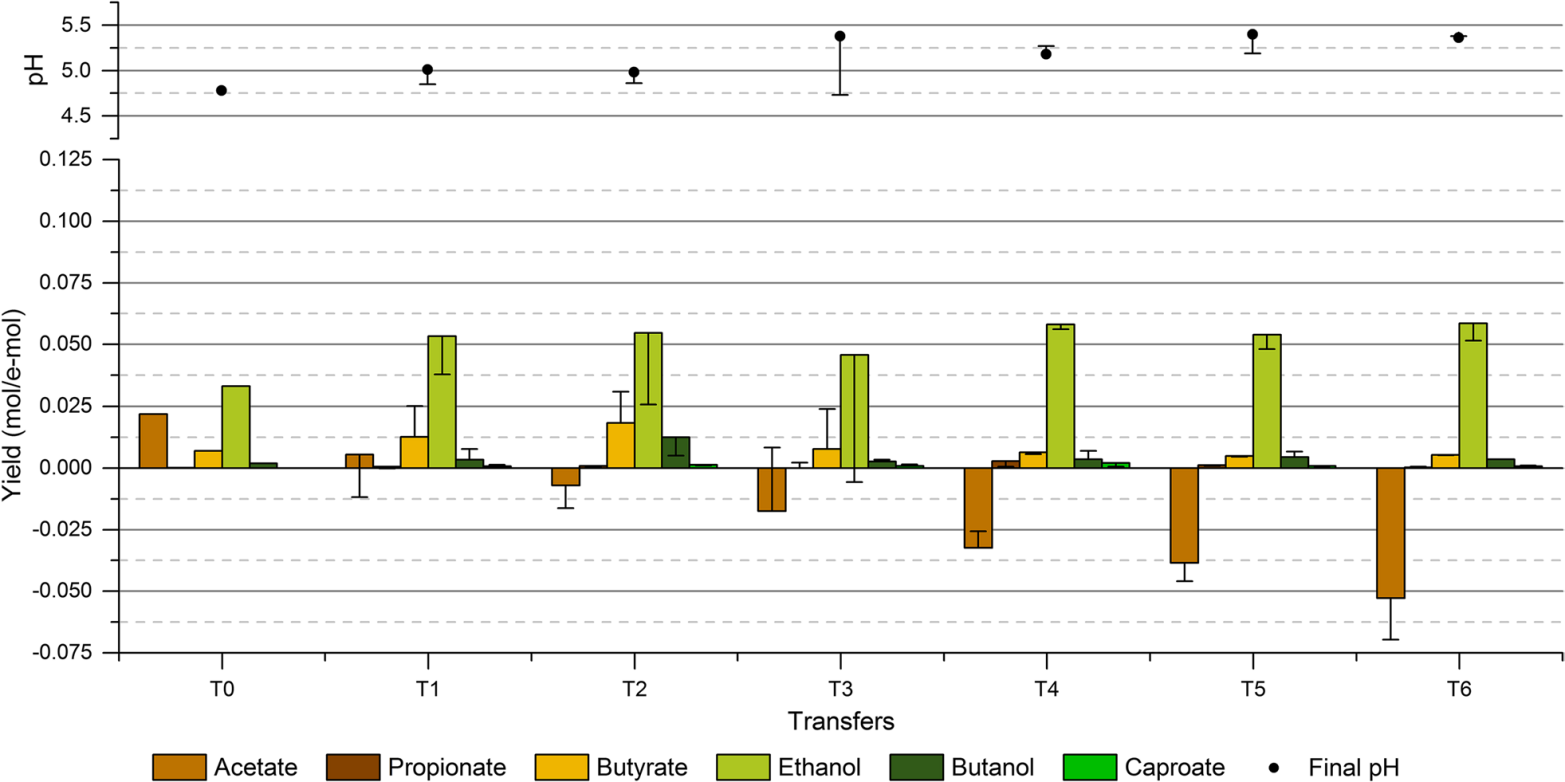
Product yields (mol/e-mol), net consumption/production of acetate (mol/e-mol of syngas consumed) and final pH for each fermentation in enrichment at pH 5 with addition of acetate (20 mM). The maximum theoretical net consumption of acetate corresponds to 0.25 mol acetate consumed/e-mol of syngas consumed. The columns show the values for the fermentation transferred and the error bars indicate the corresponding values of the duplicate experiment. Additional information on substrate consumption can be found in the Additional file 1: Figure S8

The results of the enrichment showed that the production of ethanol was enhanced by the addition of acetate. The higher ethanologenic potential of the microbial consortium at these enrichment conditions was evident as, at the first transfer, ethanol was already the main product of the fermentation and the ethanol yield was significantly higher than that of enrichment HT5YE without acetate addition (Fig. 6). As the enrichment proceeded, the ethanol production rapidly improved reaching an ethanol yield of 0.055 mol/e-mol (65.58% of the stoichiometric maximum) at transfer T2. Nevertheless, from transfer T0, both acetate and ethanol started to be used as substrates for the chain elongation reaction, resulting in increasing amounts of butyrate produced from transfer T0 to T3. Figure 6 shows the product yields obtained along the enrichment for the two replicates, where a high disparity between replicates and a high tendency for chain elongation can be observed, with butyrate even becoming the main product in one of the replicates at transfer T3. The large variations observed in transfer T3 derive from the fact that butyrate was produced through a two-step reaction, thus causing high deviations depending upon when the chain-elongation reaction started during the fermentation (fermentation profiles of transfer T3 can be found in Additional file 1: Figures S9 and S10). Although it was possible to perform the transfers while ethanol was still the main product of the fermentation in at least one of the replicates, at transfer T2 it was obvious that the chain-elongating microbial group was being enriched in the microbial consortium. Therefore, the enrichment strategy was modified from transfer T2 in an attempt to wash out the chain-elongating microbial group by transferring the cultures as soon as consumption of both CO and H_2_ started. This strategy was expected to select exclusively for carboxydotrophic microorganisms as these would be the only microbial group able to reach exponential growth phase at the moment of the transfer, favoring a gradual wash out of the chain-elongating microbial group. As expected, changing the enrichment strategy allowed reducing the chain-elongating activity of the microbial consortium, yet a complete wash out of this microbial group was not achieved since a residual amount of butyrate was still produced by the end of the enrichment. However, the decline in chain-elongating activity enabled increasing further the ethanol yields obtained during the enrichment, reaching a maximum of 0.059 mol/e-mol (70.24% of stoichiometric maximum) at transfer T6.

**Fig. 7:**
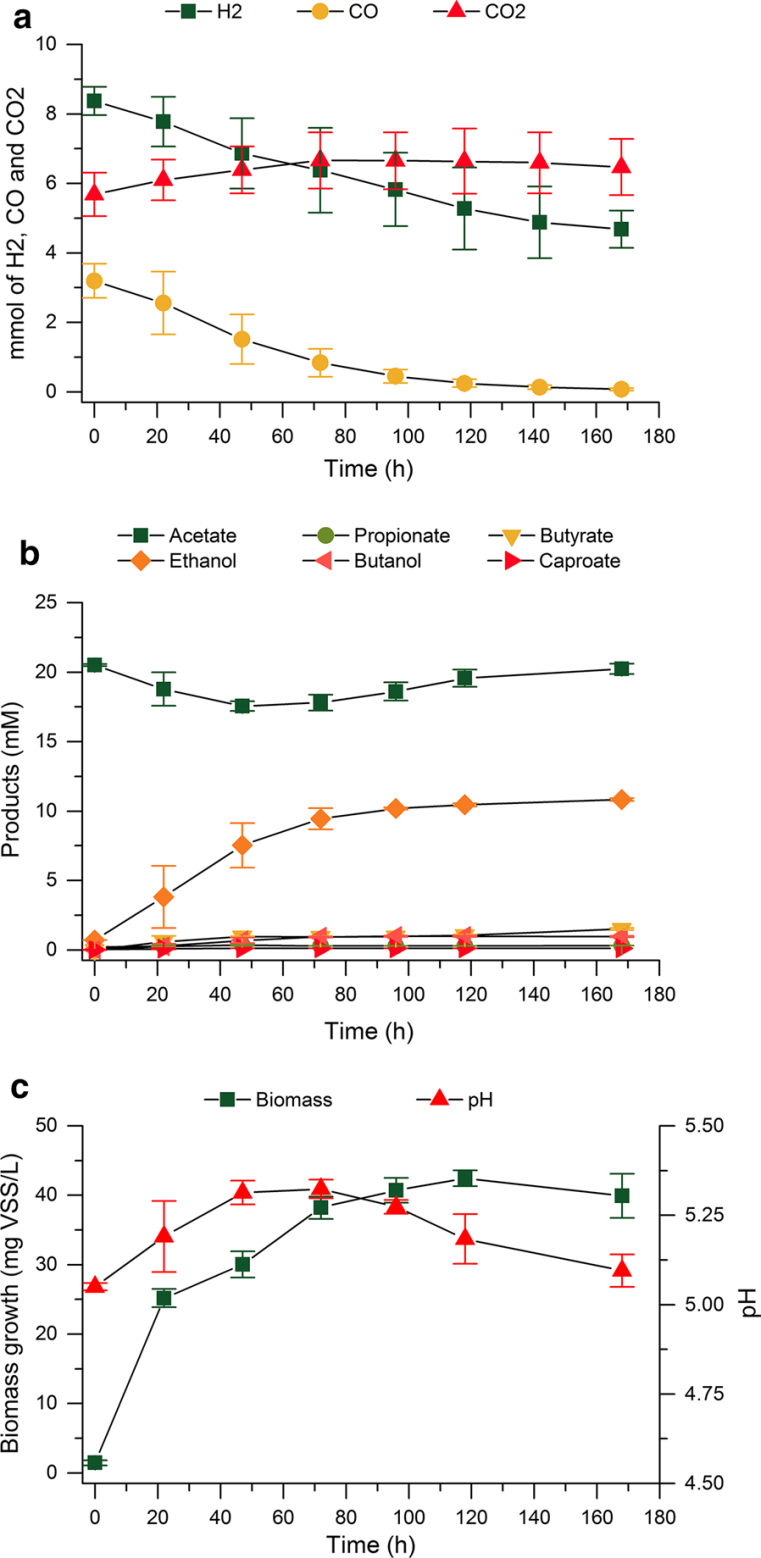
Fermentation profile of the enriched consortium HT5YE-Ac. **a** Gas composition of the headspace (mmol). **b** Concentration of products in the fermentation broth (mM). **c** Microbial growth and pH of the fermentation broth

The thermodynamic analysis predicted a much higher acetate-reducing and chain-elongating activity in the microbial consortium enriched with acetate (HT5YE-Ac) when compared to the enrichment at pH 5 (HT5YE). The higher acetate-reducing activity could be clearly observed in the fermentation profile shown in Fig. 7, where the consumption of H_2_, CO and acetate with concomitant ethanol production was apparent. This emphasizes the bioenergetic component of the metabolic shift towards ethanol, as increasing the initial acetate concentration clearly boosted the activity of acetate-reducing reactions. On the other hand, the chain elongation was expected to be inhibited by the decrease of pH along the fermentations even though this reaction would be thermodynamically favored by the addition of acetate. Nevertheless, during the course of the fermentations, the pH of the fermentation broth increased significantly as a result of the acetate conversion into ethanol (Fig. 7), which probably favored the fact that the pH inhibition of the chain-elongating activity did not operate during the enrichment. Thus, it seems that an automated pH control would be necessary to successfully prevent the chain-elongating activity through pH inhibition under these enrichment conditions. The results obtained here differed significantly from those reported by El-gammal et al. [52] since, in their study, acetate-reducing reactions were not enhanced upon addition of acetate, resulting in net acetate production at all times. However, in their study a pH of 6 and an initial acetate concentration of 13 mM was used, which reduced the effect on the ∆_r_*G*′_310 K_ for acetate-reducing reactions. In this study, the fermentation carried out by the enriched consortium HT5YE-Ac (Fig. 7) resulted in a net consumption of 0.021 ± 0.004 mol of acetate/e-mol of syngas and an ethanol yield of 0.060 ± 0.002 mol/e-mol (72.44 ± 2.11% of the stoichiometric maximum), increasing the ethanol yield obtained with the enriched consortium HT5YE by 22.49%. Similar yields, corresponding to 58.6 ± 7.4% of the stoichiometric maximum, were reported by Steinbusch et al. [22] while studying the reduction of acetate into ethanol with H_2_ as electron donor using a heat-shock-treated anaerobic sludge as inoculum. Additionally, a high chain-elongating activity was also reported in their study, where ethanol was produced in the first stage of the fermentation and was subsequently converted into butyrate.

**Fig. 8:**
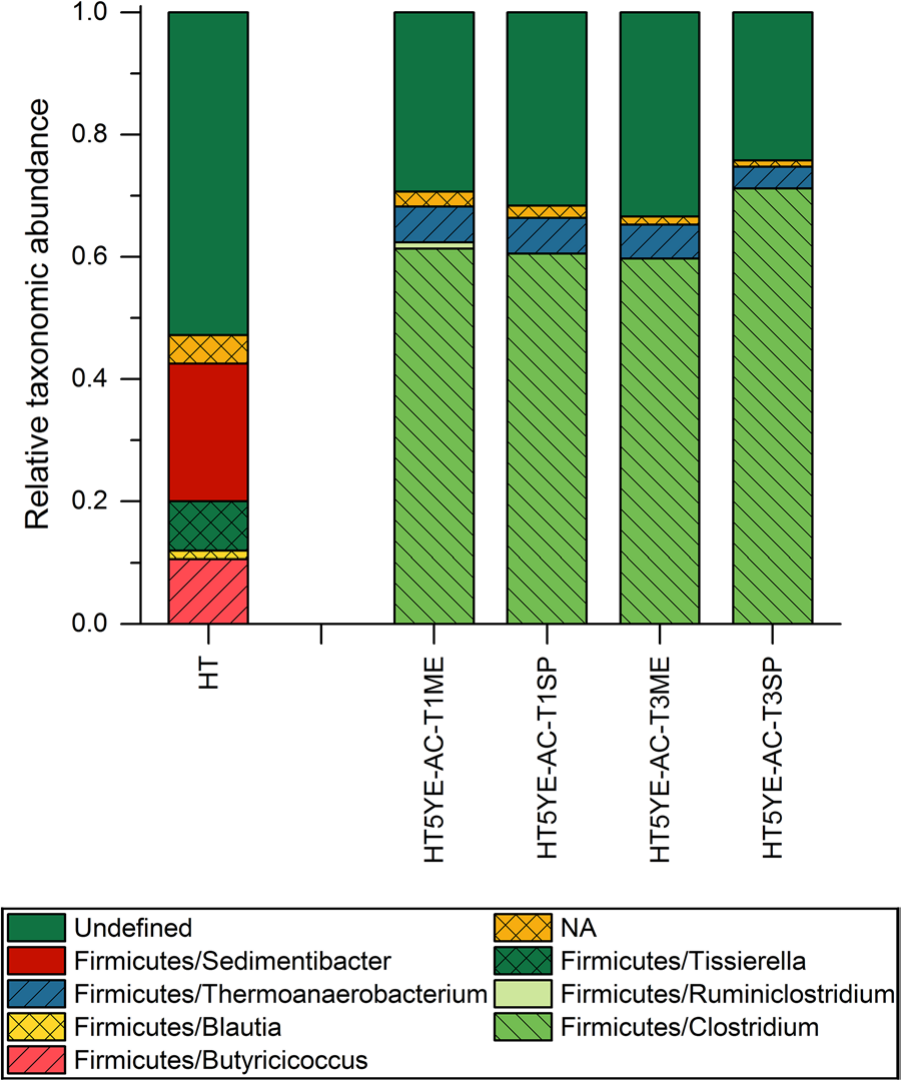
Relative taxonomic abundance of the analyzed microbial consortia in enrichment HT5YE-Ac at genus level. The label of the samples is encoded according to the enrichment name, transfer number and growth phase at the moment of the sampling. ME and SP stand for mid-exponential and stationary growth phase

The analysis of the microbial composition revealed strong similarities with the pH-based enrichment samples analyzed. In this case, the samples were withdrawn at transfers T1 and T3, which allowed evaluating the evolution of the microbial composition at an earlier stage in the enrichment. Interestingly, the results showed that the composition of the microbial community at genus level had already changed drastically at transfer T1 and remained stable until transfer T3 (Fig. 8). As in the pH-based enrichments, all enrichment samples were clearly dominated by the genus *Clostridium* with an average abundance of 63.2 ± 5.4%. However, it was not possible to identify the dominant species. There was a significant proportion of diverse OTUs (between 79.9 and 85.9% in all samples) that could not be reliably classified at this level (bootstrap value of 80% using SINTAX) (Additional file 2: Table S2). SINTAX-classified OTUs with relatively high abundance were classified as *C. nitrophenolicum* and *C. kluyveri* and were present in the enrichment samples within a range of 10.6–18.4% and 0–2.8%, respectively. This magnitude of abundances suggests a possible role during the fermentations. *C. nitrophenolicum* has never been described to consume neither CO nor H_2_. The only mention of *C. nitrophenolicum* in gas fermentation-related literature corresponds to a study on bio-H_2_-mediated production of commodity chemicals using bioelectrochemical systems, where this species had a relative abundance between 2.7% and 3.6% in the cathode biofilm [63]. In turn, *C. kluyveri* is generally referred to as the model organism for the chain elongation process in several studies using both co- and mixed cultures [51, 64]. Additionally, *C. kluyveri* was identified in an enrichment study aiming at the conversion of syngas into higher alcohols, in which this species was found to participate in the chain elongation of acetate and ethanol [21]. In this study, the relative abundance of selected OTUs corresponding to *C. kluyveri* increased from transfer T1 to T3 during the enrichment, where butyrate was observed to be produced through chain elongation (Additional file 1: Figures S9 and S10). It can be thus concluded that this species clearly contributed to the increasing chain-elongating activity observed during the early stage of this enrichment.

### Effect of enrichment conditions on microbial diversity

**Fig. 9:**
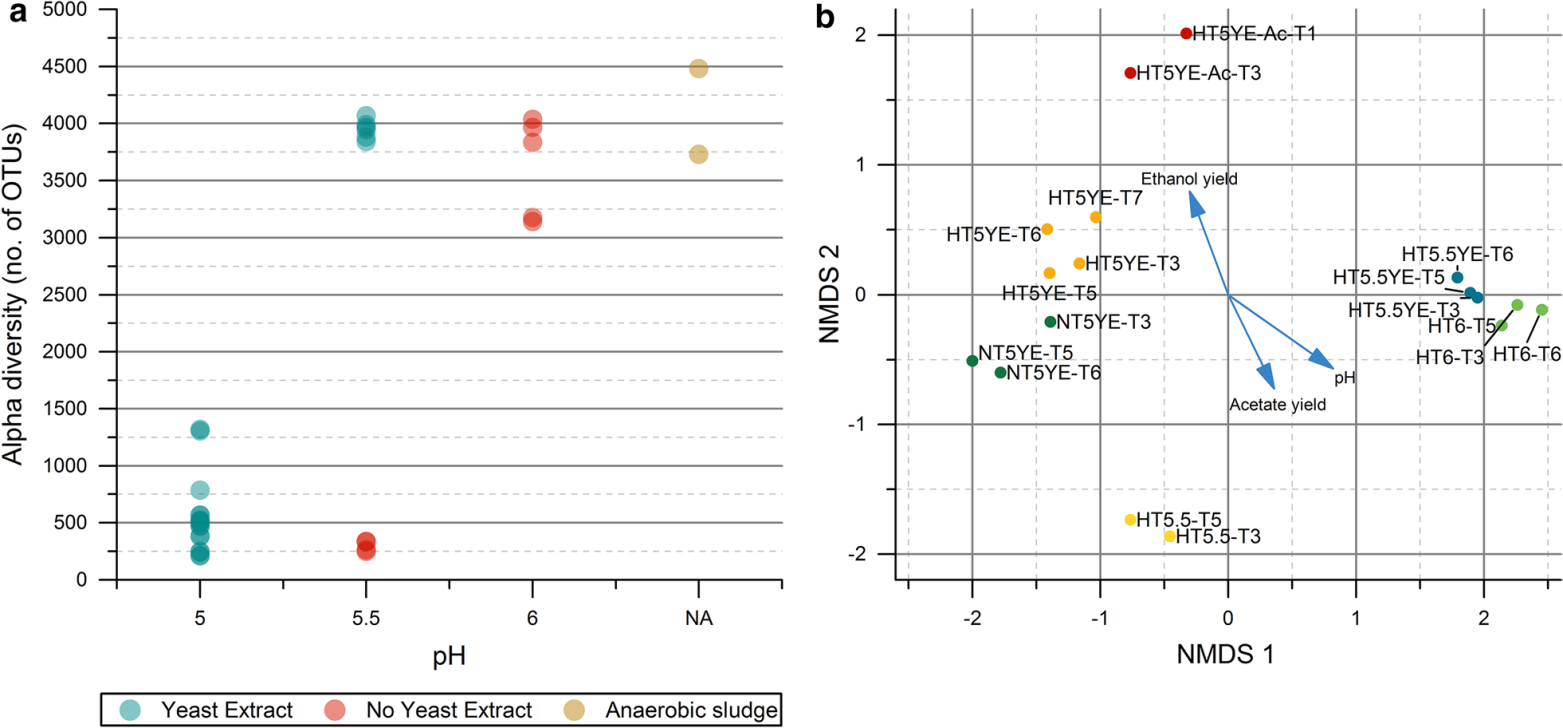
**a** Dependence of alpha diversity (measured as number of unique OTUs) on pH for all enrichment samples. NA corresponds to samples from the starting inocula. **b** Non-metric multidimensional scaling (nMDS) unconstrained ordination. The arrows represent the direction and strength of the correlation between the variables and the unconstrained ordination of samples. The label of the samples is encoded according to the enrichment series name and transfer number

Comparing the microbial diversity of all enrichment samples revealed important differences across enrichment conditions. Figure 9a shows the alpha diversity calculated for all samples sorted by initial pH conditions. The results show that both inocula used presented among the highest alpha diversity, which gradually decreased with harsher enrichment conditions. The comparison across enrichment conditions shows a clear decreasing trend in alpha diversity as the pH decreases. Although the addition of YE contributed to a higher diversity, as it can be seen by comparing the two enrichments at pH 5.5 (HT5.5 and HT5.5YE), further decreasing the pH to 5 resulted in a drastic drop in diversity despite YE addition. Thus, the pH seems to be the major factor driving the reduction of complexity observed in the enrichment cultures. Similar trends were observed when studying the microbial diversity during a hydrogenotrophic enrichment at different pH conditions using cow manure as inoculum, where it was shown that pH 5 and pH 7 sustained the lowest and the highest microbial diversity among all conditions studied [65].

The non-metric multidimensional scaling (nMDS) analysis (Fig. 9b) illustrates the degree of microbial composition similarity between enrichment samples based on their relative distance. The results show that the samples from each enrichment are grouped together as a result of their higher similarity, which indicates that the enrichment cultures had reached a stable microbial composition at transfer T3. Comparing across enrichment conditions, it can be seen that enrichments HT6 and HT5.5YE, on one hand, and enrichments HT5YE, NT5YE and HT5YE-Ac on the other, developed closely related microbial communities, although a more widespread distribution can be observed in the latter group (Fig. 9b). In turn, the microbial consortium from enrichment HT5.5 was less related to other enrichments, probably due to the drastic drop in alpha diversity as compared to HT5.5YE. A statistically significant correlation was found between the initial pH conditions of each enrichment series and their microbial composition with a *R*^2^ corresponding to 0.90 (*P* value < 0.001), which indicates that the microbial composition found in the enrichment cultures was pH-dependent (Fig. 9b). Similarly, the ethanol and acetate yields were also found to be correlated with the ordination of the samples (with a *R*^2^ of 0.73 and 0.65, respectively, *P* value < 0.001) and followed a similar gradient direction with the pH (Fig. 9b). Thus, these results show that both the microbial composition and the yield of the main products were affected by the pH conditions of each enrichment series.

## Conclusions

The enrichment strategies studied resulted in the successful selection of acetogenic bacteria from both untreated and heat-shock-treated anaerobic sludge, obtaining a number of enriched mixed microbial consortia with variable ethanologenic potential and microbial diversity as a function of the enrichment conditions applied. The composition of the microbial community was shown to shift rapidly along the enrichments, reaching a stable microbial composition dominated by the genus *Clostridium* in all cases, with a single dominant species in most of the enrichments. Both pH and nutrient supplements (YE) were found to be determinant operational parameters affecting the specific composition of the consortia and their microbial diversity. The ethanologenic potential of the enriched consortia was strongly dependent on the pH conditions applied, where an ethanol yield of 59.15 ± 0.18% of the stoichiometric maximum was achieved in pH-based enrichments at the lowest pH tested (pH 5). On the other hand, the addition of YE triggered the production of C4 compounds, opening the way for the production of MCFAs and higher alcohols. The thermodynamic approach used for the analysis of the metabolic network of reactions carried out by syngas-converting microbial consortia proved to be highly useful for assisting the design and interpretation of enrichment strategies. Based on the qualitative predictions of the thermodynamic analysis, it was possible to improve the product selectivity and enhance the maximum ethanol yield obtained in pH-based enrichments by 22.5% (72.44 ± 2.11% of the stoichiometric maximum) through an increase of the initial acetate concentration (enrichment HT5YE-Ac). Thus, this work demonstrated that a highly selective microbial activity towards the production of ethanol is possible using open-mixed microbial consortia. However, given the experimental observations made here, the ethanol yield obtained in enrichment HT5YE-Ac in batch mode cannot be extrapolated to processes in continuous mode as the long-term exposure of the enriched consortium to elevated ethanol and acetate concentrations would likely promote a high chain-elongating activity, lowering the product selectivity towards ethanol. Further work in this area is still needed to develop operational strategies able to control the chain-elongation reaction in syngas-converting microbial consortia.

## Additional files

**Additional file 1: Table S1.** t-test for comparing the biomass yields of the enrichment experiments. **Table S2.** t-test for comparing the production efficiency of the enriched consortia. **Table S3.** Metabolite production in control experiments. **Figures S1–S4.** Fermentation profiles from enrichment cultures HT5.5 and HT5.5YE. **Figure S5.** Biomass yield and substrate consumption in enrichment experiments. **Figures S6, S7.** Fermentation profiles from enrichment cultures HT5.5YE and HT5YE. **Figure S8.** biomass yield and substrate consumption in enrichment HT5YE-Ac. **Figures S9, S10.** Fermentation profiles from enrichment HT5YE-Ac at transfer T3.

**Additional file 2.** **Tables S1, S2.** Relative taxonomic abundance at genus and species level for initial inocula and enrichment samples. **Table S3.** Normalized read counts mapping in all samples to Operational Taxonomic Units (OTUs) and their taxonomy classification.
