# Supplementary material for: Enrichment of syngas-converting mixed microbial consortia for ethanol production and thermodynamics-based design of enrichment strategies
Source: Biotechnol Biofuels. 2018 Jul 19;11:198. doi: 10.1186/s13068-018-1189-6 (PMC6052697; doi:10.1186/s13068-018-1189-6)
Supplement: Supplementary file 1 — Additional file 1: Table S1. t-test for comparing the biomass yields of the enrichment experiments. Table S2. t-test for comparing the production efficiency of the enriched consortia. Table S3. Metabolite production in control experiments. Figures S1–S4. Fermentation profiles from enrichment cultures HT5.5 and HT5.5YE. Figure S5. Biomass yield and substrate consumption in enrichment experiments. Figures S6, S7. Fermentation profiles from enrichment cultures HT5.5YE and HT5YE. Figure S8. biomass yield and substrate consumption in enrichment HT5YE-Ac. Figures S9, S10. Fermentation profiles from enrichment HT5YE-Ac at transfer T3. [file 13068_2018_1189_MOESM1_ESM.docx]

Enrichment of syngas-converting mixed microbial consortia for ethanol production and thermodynamics-based design of enrichment strategies – Additional file 1.

Table S1. Average apparent biomass yield for enrichment experiments from transfer T3 to T6 and p-values from t-test analysis.

|  | Biomass yield  (mg VSS/e-mol) | P-value | HT6 | HT5.5 | HT5.5YE | HT5YE | NT5YE |
| --- | --- | --- | --- | --- | --- | --- | --- |
| HT6 | 0.216±0.042 | HT6 |  |  |  |  |  |
| HT5.5* | 0.168±0.040 | HT5.5 | 0.121 |  |  |  |  |
| HT5.5YE | 0.221±0.026 | HT5.5YE | 0.844 | 0.035 |  |  |  |
| HT5YE | 0.279±0.071 | HT5YE | 0.077 | 0.016 | 0.054 |  |  |
| NT5YE | 0.262±0.036 | NT5YE | 0.110 | 0.002 | 0.039 | 0.563 |  |

*Average apparent biomass yield of enrichment series HT5.5 was calculated using values from transfer T3 to T5.

Table S2. Average production efficiency calculated in Cmol and e-mol recovery, and p-values from t-test analysis.

|  |  | Production  efficiency (%) | P-value | | HT6 | HT5.5 | HT5.5YE | HT5YE | NT5YE |
| --- | --- | --- | --- | --- | --- | --- | --- | --- | --- |
| HT6 | Cmol | 83.16±0.70% | HT6 | Cmol |  |  |  |  |  |
|  | e-mol | 92.92±0.54% |  | e-mol |  |  |  |  |  |
| HT5.5 | Cmol | 77.33±2.31% | HT5.5 | Cmol | 0.017 |  |  |  |  |
|  | e-mol | 85.83±2.46% |  | e-mol | 0.012 |  |  |  |  |
| HT5.5YE | Cmol | 93.18±1.69% | HT5.5YE | Cmol | 0.007 | 0.000 |  |  |  |
|  | e-mol | 89.84±1.80% |  | e-mol | 0.128 | 0.090 |  |  |  |
| HT5YE | Cmol | 80.29±0.83% | HT5YE | Cmol | 0.115 | 0.133 | 0.003 |  |  |
|  | e-mol | 88.57±1.77% |  | e-mol | 0.234 | 0.334 | 0.618 |  |  |
| NT5YE | Cmol | 91.60±6.41% | NT5YE | Cmol | 0.203 | 0.077 | 0.764 | 0.126 |  |
|  | e-mol | 95.01±3.29% |  | e-mol | 0.464 | 0.035 | 0.144 | 0.115 |  |

*Production efficiency of HT5.5 calculated using data from transfer T4 and T5 of enrichment experiments.

Table S3. Net production/consumption in control experiments and total Cmol (Cmmol) and e-mol (e-mmol) production.

|  | Acetate  (mmol) | Propionate  (mmol) | Butyrate  (mmol) | Ethanol  (mmol) | Butanol  (mmol) | Caproate  (mmol) | Total  Cmmol | Total  e-mmol |
| --- | --- | --- | --- | --- | --- | --- | --- | --- |
| HT6 | 0.004 | 0.000 | 0.000 | 0.000 | 0.000 | 0.000 | 0.007 | 0.029 |
| HT5.5 | 0.024 | 0.000 | 0.000 | -0.005 | 0.000 | 0.000 | 0.039 | 0.137 |
| HT5.5YE | 0.332 | 0.018 | 0.020 | -0.109 | -0.020 | 0.000 | 0.496 | 1.498 |
| HT5YE | 0.066 | 0.013 | 0.037 | -0.038 | 0.000 | -0.002 | 0.230 | 0.926 |
| NT5YE | 0.324 | 0.027 | 0.024 | -0.110 | -0.019 | 0.013 | 0.606 | 2.088 |


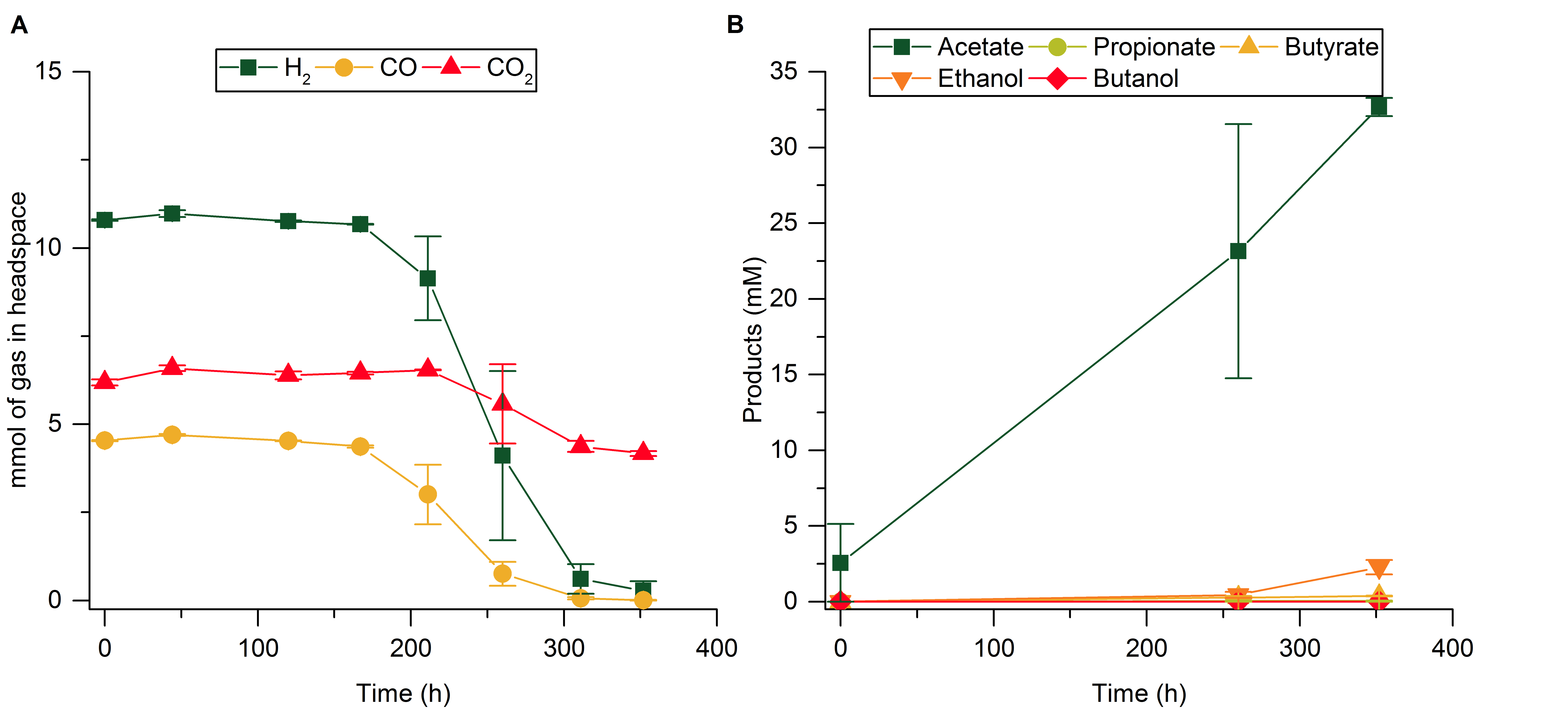


Figure S1. Fermentation profile of enrichment HT5.5 at transfer T1 (average of duplicates). A Gas composition of the headspace (mmol). B Concentration of products in the fermentation broth (mM) and microbial growth (mg VSS/L).


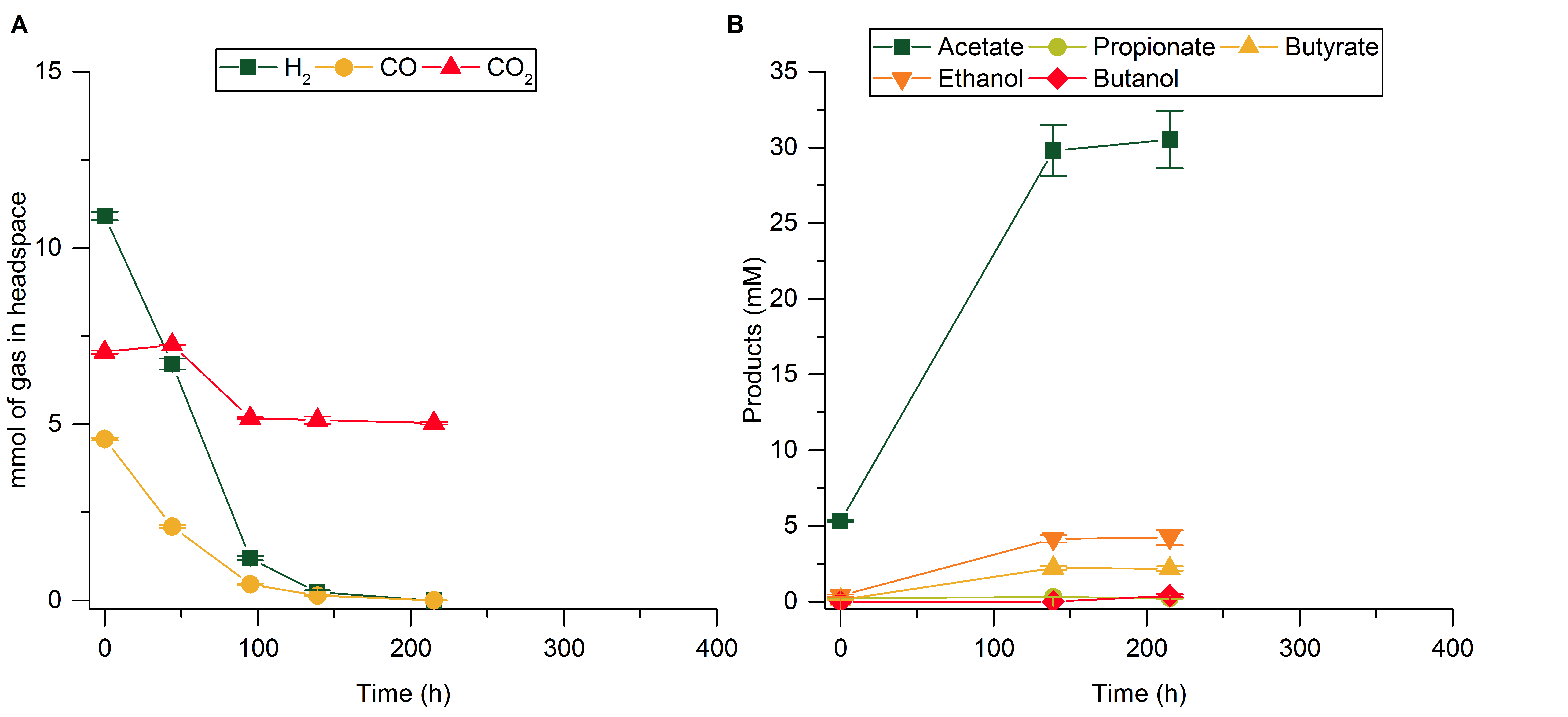


Figure S2. Fermentation profile of enrichment HT5.5YE at transfer T1 (average of duplicates). A Gas composition of the headspace (mmol). B Concentration of products in the fermentation broth (mM) and microbial growth (mg VSS/L).


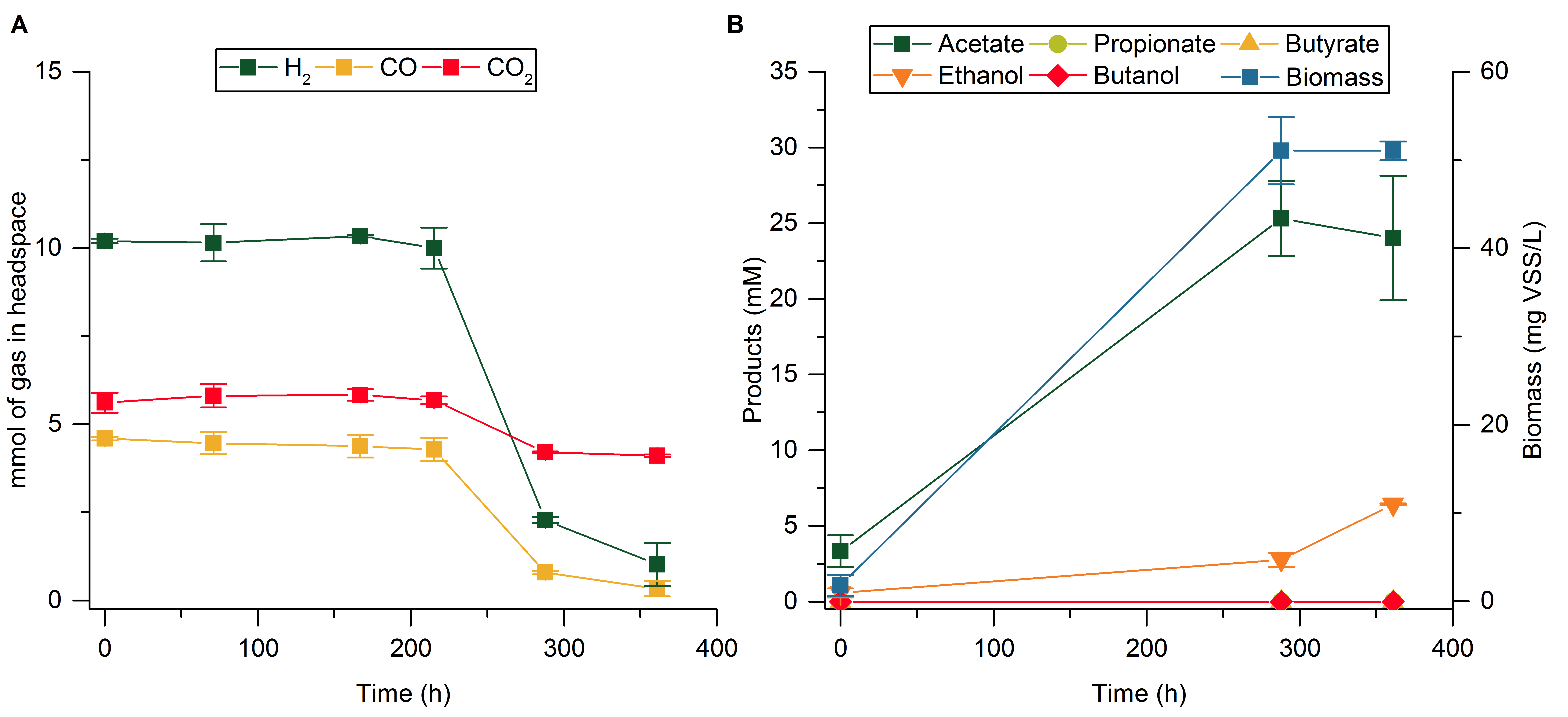


Figure S3. Fermentation profile of enrichment HT5.5 at transfer T4 (average of duplicates). A Gas composition of the headspace (mmol). B Concentration of products in the fermentation broth (mM) and microbial growth (mg VSS/L).


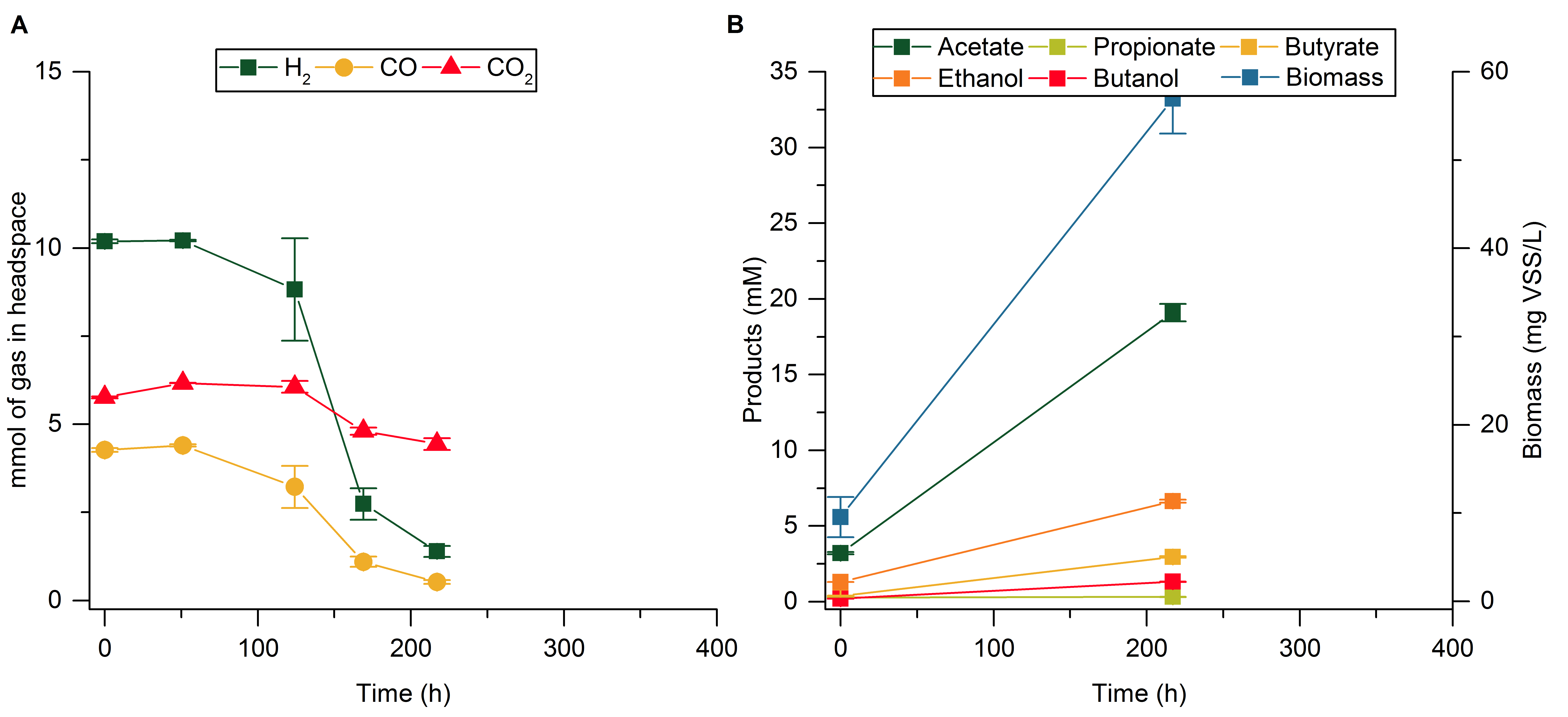


Figure S4. Fermentation profile of enrichment HT5.5YE at transfer T4 (average of duplicates). A Gas composition of the headspace (mmol). B Concentration of products in the fermentation broth (mM) and microbial growth (mg VSS/L).





Figure S5. Apparent biomass yield in enrichment experiments from transfer T3, and percentage of H_2_ and CO conversion for each batch experiment. The columns show the values for the fermentation transferred and the error bars indicate the corresponding values of the duplicate experiment. A Enrichment HT6 at an initial pH of 6; B Enrichment HT5.5 at an initial pH of 5.5; C Enrichment HT5.5YE at an initial pH of 5.5 with YE (0.5 g/L); D Enrichment HT5YE at an initial pH of 5 with YE (0.5 g/L); E Enrichment NT5YE at an initial pH of 5 with YE (0.5 g/L).


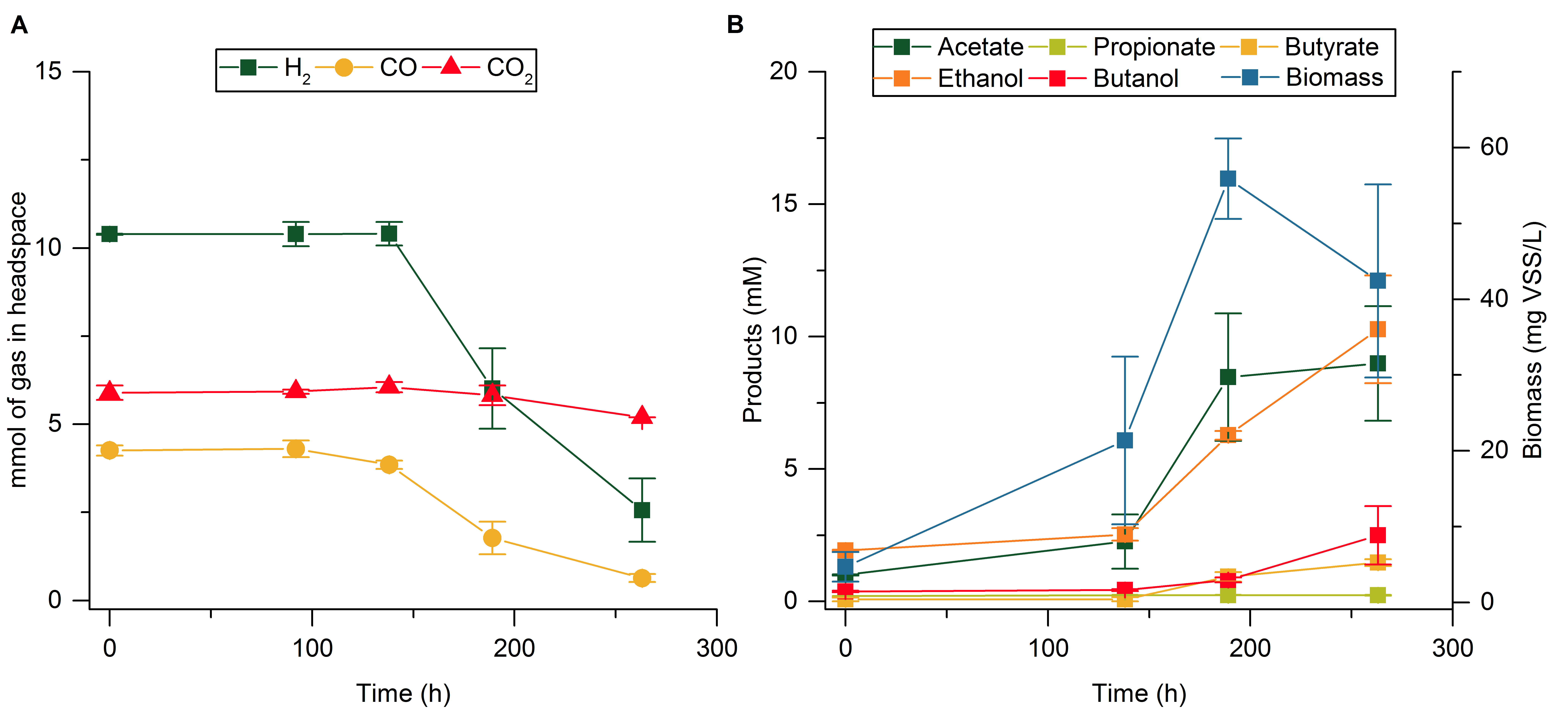


Figure S6. Fermentation profile of enrichment HT5YE at transfer T4 (average of duplicates). A Gas composition of the headspace (mmol). B Concentration of products in the fermentation broth (mM) and microbial growth (mg VSS/L).


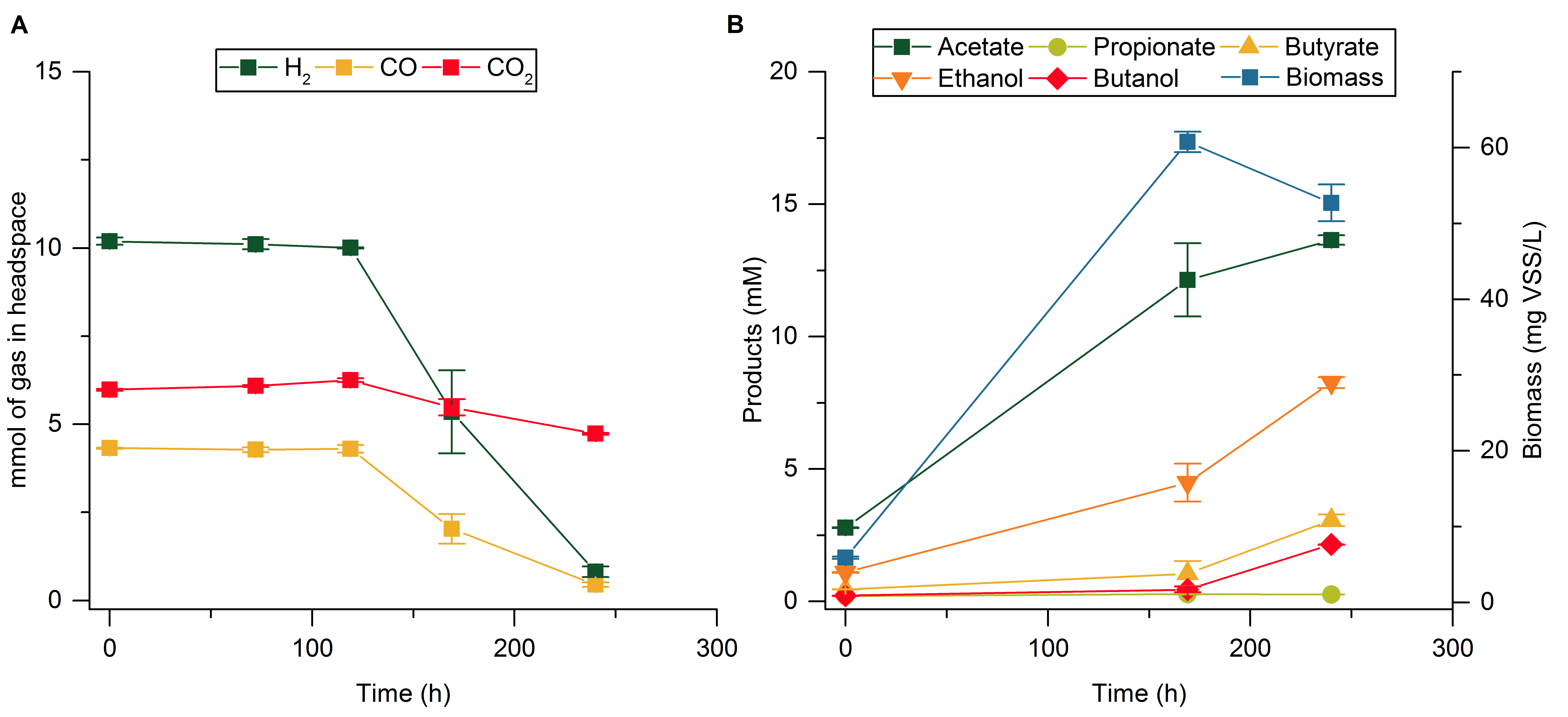


Figure S7. Fermentation profile of enrichment HT5.5YE at transfer T5 (average of duplicates). A Gas composition of the headspace (mmol). B Concentration of products in the fermentation broth (mM) and microbial growth (mg VSS/L).


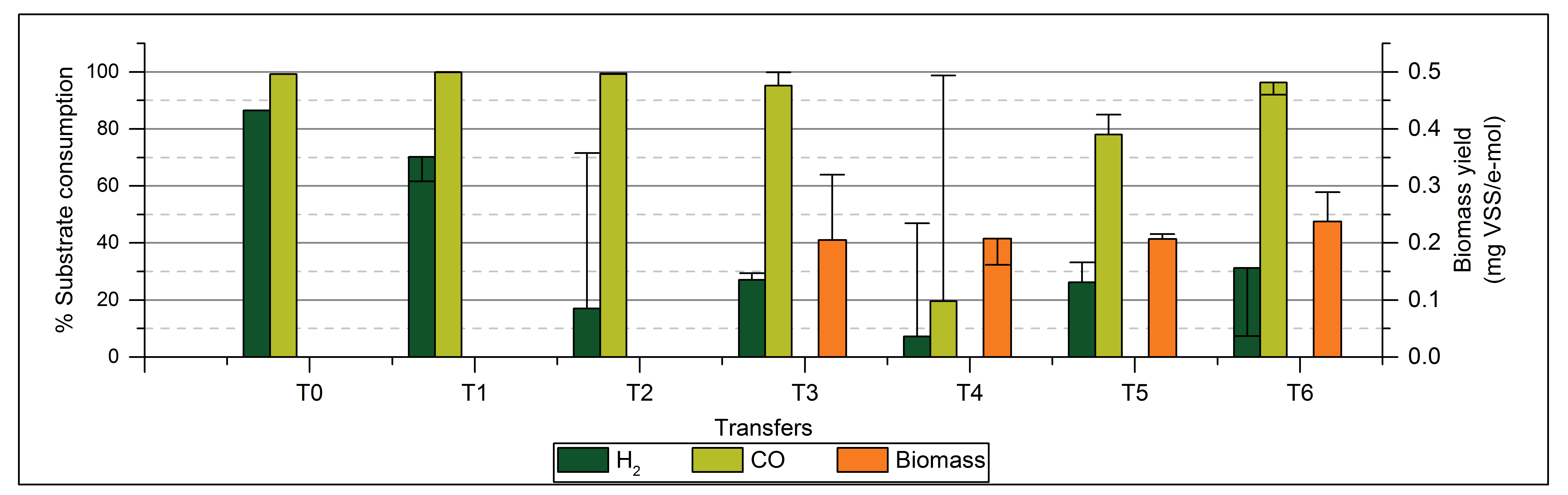


Figure S8. Apparent biomass yield for enrichment HT5YE-Ac from transfer T3, and percentage of H_2_ and CO conversion. The columns show the values for the fermentation transferred and the error bars indicate the corresponding values of the duplicate experiment.


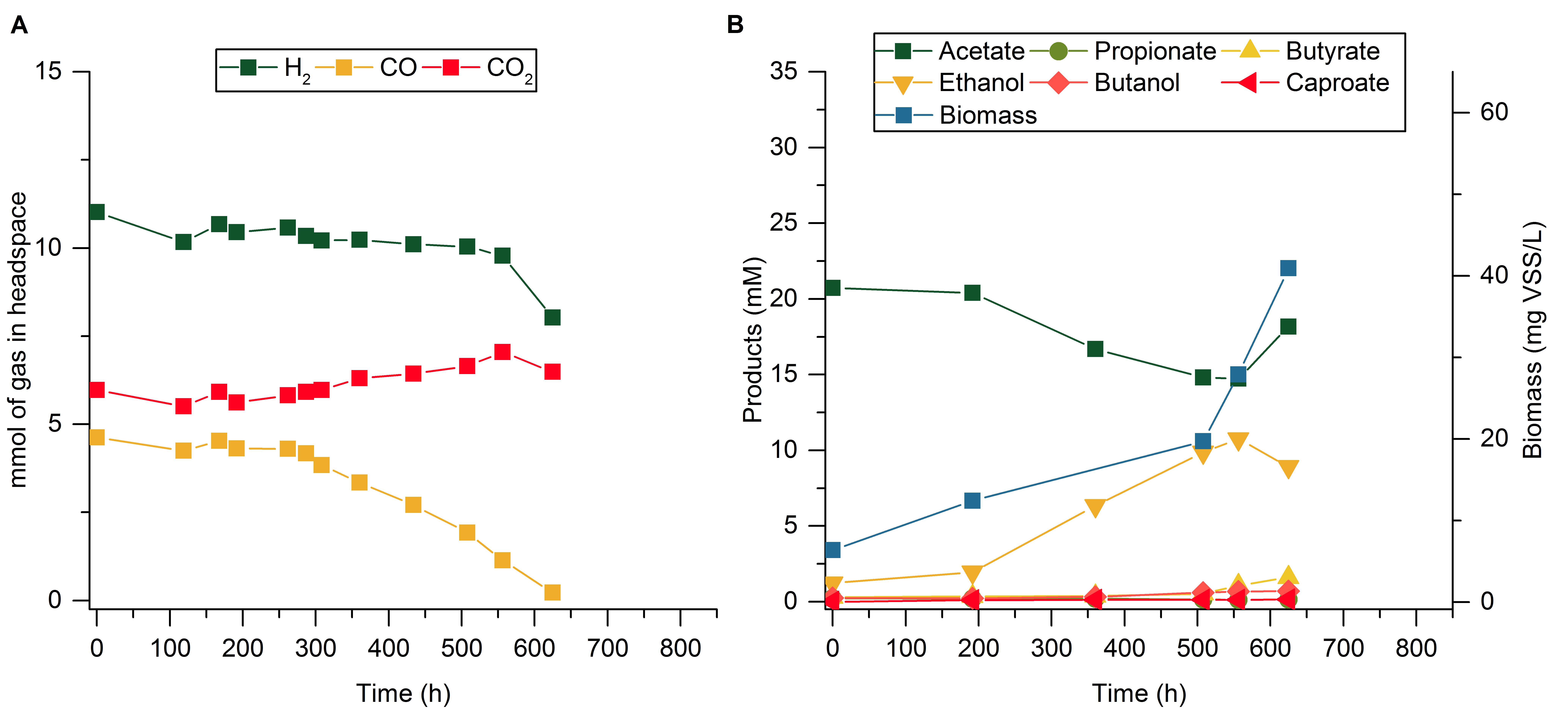


Figure S9. Fermentation profile of enrichment HT5YE-Ac at transfer T3 (replicate 1). A Gas composition of the headspace (mmol). B Concentration of products in the fermentation broth (mM) and microbial growth (mg VSS/L).


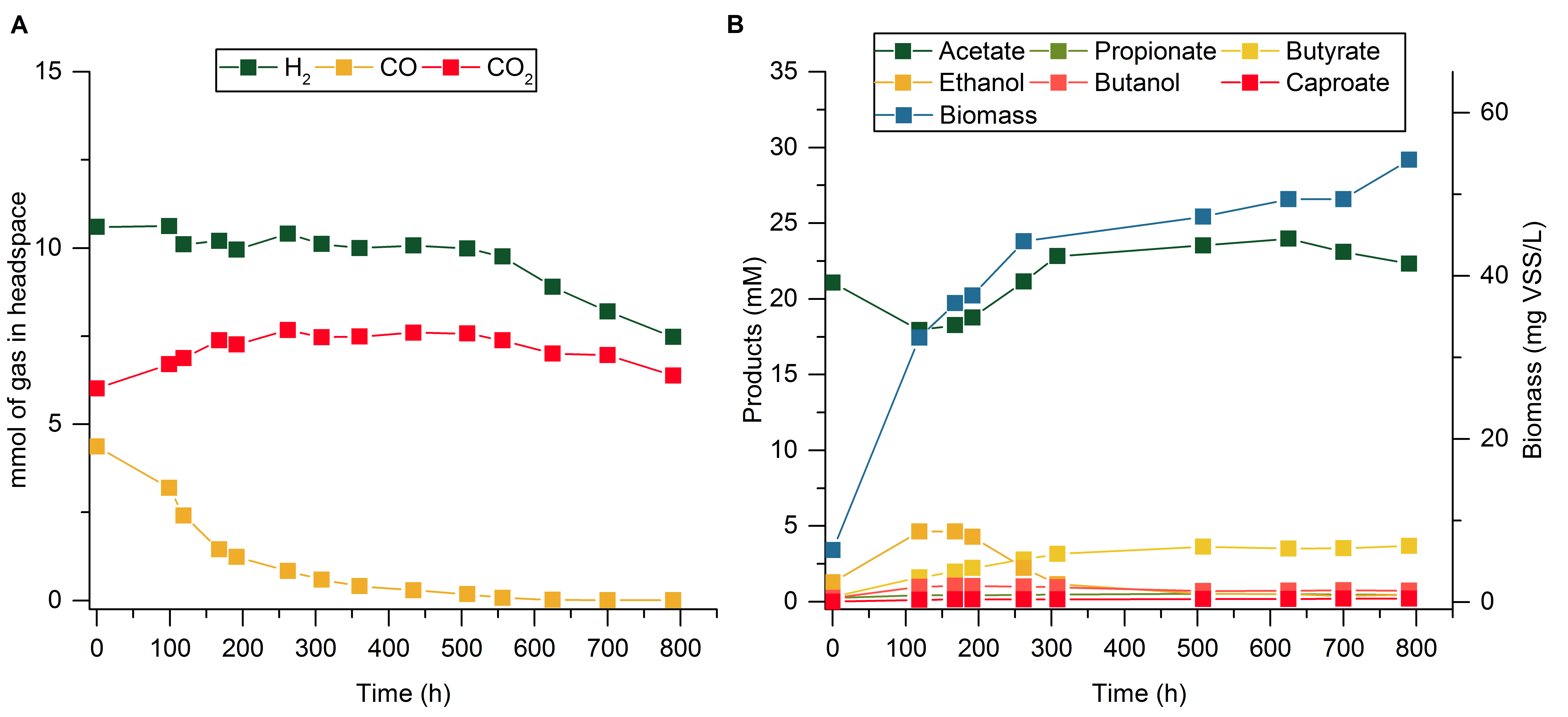


Figure S10. Fermentation profile of enrichment HT5YE-Ac at transfer T3(replicate 2). A Gas composition of the headspace (mmol). B Concentration of products in the fermentation broth (mM) and microbial growth (mg VSS/L).
